# Transcriptional Control of Glutaredoxin *GRXC9* Expression by a Salicylic Acid-Dependent and NPR1-Independent Pathway in *Arabidopsis*

**DOI:** 10.1007/s11105-014-0782-5

**Published:** 2014-08-14

**Authors:** Ariel Herrera-Vásquez, Loreto Carvallo, Francisca Blanco, Mariola Tobar, Eva Villarroel-Candia, Jesús Vicente-Carbajosa, Paula Salinas, Loreto Holuigue

**Affiliations:** 1 Departamento de Genética Molecular y Microbiología, Facultad de Ciencias Biológicas, Pontificia Universidad Católica de Chile, Alameda 340, Santiago, Chile; 2 Centro de Biotecnología y Genómica de Plantas (UPM-INIA), Universidad Politécnica de Madrid, 28223 Pozuelo de Alarcón, Madrid, Spain

**Keywords:** *as*-*1-*like element, Glutaredoxin *GRXC9* (*GRX480*), NPR1-independent, Salicylic acid, TGA transcription factors

## Abstract

Salicylic acid (SA) is a key hormone that mediates gene transcriptional reprogramming in the context of the defense response to stress. *GRXC9*, coding for a CC-type glutaredoxin from *Arabidopsis*, is an SA-responsive gene induced early and transiently by an NPR1-independent pathway. Here, we address the mechanism involved in this SA-dependent pathway, using *GRXC9* as a model gene. We first established that *GRXC9* expression is induced by UVB exposure through this pathway, validating its activation in a physiological stress condition. *GRXC9* promoter analyses indicate that SA controls gene transcription through two *activating sequence*-*1* (*as*-*1*)-like elements located in its proximal region. TGA2 and TGA3, but not TGA1, are constitutively bound to this promoter region. Accordingly, the transient recruitment of RNA polymerase II to the *GRXC9* promoter, as well as the transient accumulation of gene transcripts detected in SA-treated WT plants, was abolished in a *knockout* mutant for the TGA class II factors. We conclude that constitutive binding of TGA2 is essential for controlling *GRXC9* expression, while binding of TGA3 in a lesser extent contributes to this regulation. Finally, overexpression of *GRXC9* indicates that the *GRXC9* protein negatively controls its own gene expression, forming part of the complex bound to the *as*-*1*-containing promoter region. These findings are integrated in a model that explains how SA controls transcription of *GRXC9* in the context of the defense response to stress.

**Electronic supplementary material:**

The online version of this article (doi:10.1007/s11105-014-0782-5) contains supplementary material, which is available to authorized users.

## Introduction

Salicylic acid (SA) is a key plant hormone involved in stress defense responses against a wide range of biotrophic and hemibiotrophic pathogens and abiotic stress conditions such as UV, high light radiation, ozone exposure, salinity, osmotic, and drought stress (Borsani et al. 2001; Garcion et al. 2008; Lee et al. 2010; Mateo et al. 2006; Miura et al. 2013; Wildermuth et al. 2001; Nawrath and Metraux 1999; Ogawa et al. 2005). In response to stress, SA triggers a global transcriptional reprogramming in the infected/damaged tissues, as well as in the neighboring cells, orchestrating local and systemic defense responses (Vlot et al. 2009; Fu and Dong 2013).

Recent reports support evidence that SA interplays with redox signals, such as H_2_O_2_ and glutathione, in the modulation of the defense response (Foyer and Noctor 2011; Dubreuil-Maurizi and Poinssot 2012; Noshi et al. 2012; Han et al. 2013). Although this interplay is being increasingly recognized (Fu and Dong 2013), the precise mechanisms that govern this relationship are still unknown. We previously reported that a subset of the early SA-inducible genes (early SAIGs) code for enzymes with glutathione (GSH)-dependent antioxidant and detoxifying activities, such as glutaredoxins (*GRX*) and glutathione *S*-transferases (*GST*) (Blanco et al. 2005, 2009). Moreover, we showed that the expression of *GRXS13*, one of the *GRXs* coded by early SAIGs, is critical for limiting basal and high light stress-induced reactive oxygen species (ROS) production and for regulation of the ascorbate/dehydroascorbate (ASC/DHA) ratio after stress (Laporte et al. 2012). These data support the idea that these genes could be involved in the ROS-scavenging/antioxidant network that contains the oxidative burst produced under stress conditions.

Here, we studied *GRXC9* (also known as *GRX480*), a second *GRX* gene identified as an early SAIG (Blanco et al. 2009). The *GRXS13* and *GRXC9* genes code for glutaredoxins belonging to the plant-specific CC-type in *Arabidopsis* (Ndamukong et al. 2007; La Camera et al. 2011; Laporte et al. 2012). Glutaredoxins are small disulfide oxidoreductases that catalyze the reduction of disulfide bridges and protein–GSH adducts (*S*-glutathionylated proteins) using the reducing power of GSH and NADPH (Rouhier et al. 2008). In this work, we specifically focus in unrevealing the transcriptional control mechanisms of *GRXC9* by SA.

Transcriptional activation of the majority of the SAIGs, including the pathogenesis-related 1 gene (*PR*-*1* gene, the most frequently used marker gene for SA signaling) is mediated by the master coactivator nonexpressor of pathogenesis-related genes 1 (NPR1) (Fu and Dong 2013; Dong 2004). SA controls the nuclear targeting and activity of the NPR1 protein, via posttranslational modifications and degradation (Fu et al. 2012; Mou et al. 2003; Tada et al. 2008). Recently, the NPR1 paralogs NPR3 and NPR4 were identified as direct receptors of SA that regulate NPR1 degradation, controlling in this way the SA responses mediated by this coactivator (Fu et al. 2012). On the other hand, there is a second pathway that leads to the early and transient activation of SAIGs, via a NPR1-independent mechanism, as we and other groups have previously reported (Lieberherr et al. 2003; Uquillas et al. 2004; Blanco et al. 2005, 2009; Fode et al. 2008; Langlois-Meurinne et al. 2005; Shearer et al. 2012). The mechanism by which SA activates the expression of these early SAIGs is still unknown. In this work, we assess this mechanism using *GRXC9* as a model for the SA-dependent and NPR1-independent pathway that controls defense gene expression.

Promoter analyses of early SAIGs show overrepresentation of a *cis*-*acting* element with high homology to the *activating sequence*-*1* (*as*-*1*) (Blanco et al. 2005, 2009). The *as*-*1* element consists of two adjacent variants of the palindromic sequence TGAC/GTCA (TGACG box), separated by four base pairs (Ellis et al. 1993; Krawczyk et al. 2002). The *as*-*1* element was first identified in the Cauliflower Mosaic Virus 35S (CaMV 35S) promoter, as an element that conferred basal expression in root tips (Benfey et al. 1989). Subsequent studies showed that the *as*-*1* element from the CaMV 35S promoter responds early and transiently to SA (Qin et al. 1994), to xenobiotic compounds like the synthetic auxin 2,4-dichlorophenoxyacetic acid (2,4D) (Johnson et al. 2001), and to H_2_O_2_ and methyl viologen (Garreton et al. 2002). Accordingly, this particular array of two adjacent TGACG boxes has been found overrepresented, not only in early SAIGs promoters (Blanco et al. 2005, 2009) but also in the promoters of plant genes associated to chemical detoxification process induced by xenobiotic chemicals like 2,4-D and oxidized lipids (oxilipins) (Fode et al. 2008; Köster et al. 2012; Johnson et al. 2001).

TGACG boxes are recognized by basic leucine zipper (bZIP) factors of the TGA family, which has 10 members in *Arabidopsis* (Jakoby et al. 2002; Gatz 2012). Seven of these factors have been associated to the defense response (TGA1-TGA7), being class II (TGA2, TGA5, and TGA6) the most relevant for the SA pathway (Gatz 2012). In fact, involvement of TGA class II factors in the canonic pathway that controls expression of SA- and NPR1-dependent genes containing TGA motifs in their promoters has been extensively reported using the *Arabidopsis PR*-*1* gene as a model (Lebel et al. 1998; Kesarwani et al. 2007). In contrast, the pathway that activates SA-dependent and NPR1-independent genes containing the *as*-*1*-like element in their promoters has been far less explored.

In this work, we show evidence that *GRXC9* expression is activated by stress, such as UVB radiation, via an SA-dependent and NPR1-independent pathway. Bioinformatics analysis of the *GRXC9* promoter indicates the presence of two putative *as*-*1*-like elements in its proximal region. We show that both elements are functionally relevant and required for the SA-mediated induction of the gene. Moreover, our data indicate that TGA2 and TGA3, but not TGA1, are constitutively bound to the *GRXC9* promoter in vivo. We also confirm the requirement of TGA class II factors for the recruitment of the RNA polymerase II (Pol II) enzyme to the basal promoter and for the transcriptional induction of the gene upon SA stimuli. Analysis of the transactivation as well as the homodimerization and heterodimerization ability of TGA class II and TGA3 factors gives further insights into the mechanism of induction of antioxidant genes by the SA/TGA2-3/*as*-*1*-like pathway. Interestingly, we show evidence indicating that *GRXC9* participates in the control of the expression of its own gene, suggesting its involvement in the transient activation of early SAIGs. Physiological and mechanistic implications of these findings are discussed.

## Results

### *GRXC9* Gene Expression Is Induced by UVB Exposure, via an SA-Dependent and NPR1-Independent Mechanism

We previously reported that *GRXC9* gene expression is early and transiently induced by exogenous treatment with SA, via an NPR1-independent pathway (Blanco et al. 2009). We confirmed these results by quantitative reverse transcription PCR (RT-qPCR), showing that a significant increase in *GRXC9* transcript levels occurs after 2.5 h of SA treatment in both WT and *npr1*-*1* mutant plants (Online Resource 1). Considering that these results are in disagreement with a previous report that claims that *GRXC9* expression induced by SA is dependent on NPR1 (Ndamukong et al. 2007), we used the *npr3*-*1 npr4*-*3* double mutant to further assess this point. NPR3 and NPR4 proteins are required to control NPR1 degradation (Fu et al. 2012). The *npr3*-*1 npr4*-*3* mutant shows increased basal levels of NPR1 protein and, as a consequence, higher basal expression levels of NPR1-dependent *PR1*, *PR2*, and *PR5* genes than the WT plants (Fu et al. 2012; Zhang et al. 2006). In the case of *GRXC9* expression, we detected an early and transient increase of transcript levels in the *npr3 npr4* double mutants (Online Resource 1), while the basal levels remained unchanged when compared to WT or *npr1*-*1* plants (Online Resource 1, insert). This evidence confirms that *GRXC9* gene expression induced by SA treatment does not depend on NPR1 levels.

**Fig. 1:**
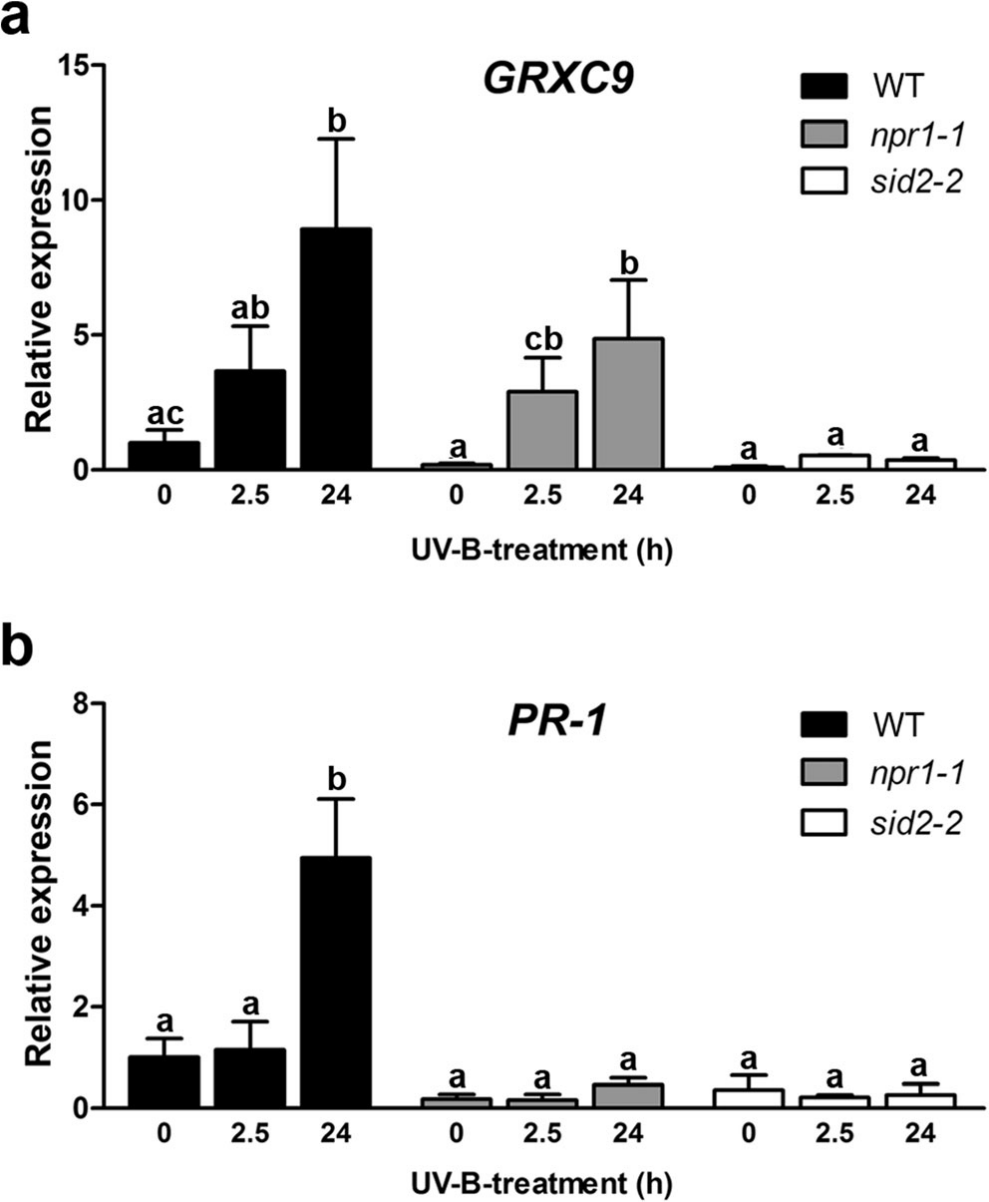
*GRXC9* expression levels in WT, *sid2*-*2*, and *npr1*-*1* seedlings upon UVB chronic exposure. Expression levels of *GRXC9* (**a**) and *PR1* (**b**) genes in 15-day-old seedlings from WT (*black bars*), *npr1*-*1* (*gray bars*), and *sid2*-*2* (*white bars*) backgrounds were exposed to UVB light. The transcript levels for each gene were quantified by RT-qPCR from samples collected after 0, 2.5, and 24 h of UVB exposure. The relative expression was calculated by normalizing the *GRXC9* and *PR1* transcript levels to that of the *YLS8* gene and to the WT basal levels. *Error bars* represent the mean ± standard error from 3 to 6 replicates. Data from a representative replicate is shown in Online Resource 2. *Letters* above the *bars* indicate significant differences based on unpaired *t* test (*n* ≥ 3, *p* < 0.05)

To further validate the SA-dependent and NPR1-independent expression of *GRXC9* under physiological stress, we used UVB radiation as a stress condition since SA has been identified as a signaling molecule in this defense response (Surplus et al. 1998). To evaluate the *GRXC9* gene expression dependence on SA, we used *sid2*-*2* mutant plants that are deficient in SA biosynthesis (Wildermuth et al. 2001). Then, *GRXC9* transcript levels were measured by RT-qPCR in WT, *npr1*-*1* and *sid2*-*2 Arabidopsis* plants exposed for 2.5 and 24 h to UVB light. Mean values from 3 to 6 replicates are shown in Fig. 1, while data from a representative replicate are shown in Online Resource 2. We detected significant increase in *GRXC9* transcript levels after 24 h of stress exposure in WT and *npr1*-*1* plants. This response was almost completely abolished in the *sid2*-*2* mutant plants (Fig. 1a, Online Resource 2a). *PR*-*1* gene expression after UVB treatment was evaluated as a control for the SA- and NPR1-dependent pathway (Fig. 1b, Online Resource 2b).

Concerning the basal levels of *GRXC9* and *PR*-*1* expression, we detected a reduction in *npr1*-*1* and *sid2*-*2* mutants compared to WT plants, but these differences were not statistically significant (Fig. 1a, b, Online Resource 1 and 2). Together, these results show that *GRXC9* is an SA-dependent and NPR1-independent stress responsive gene. We used the *GRXC9* gene as a model to inquire about the mechanism involved in this pathway.

### Two *as*-*1*-Like Elements in the *GRXC9* Promoter Are Required and Sufficient for SA-Mediated Transcriptional Activation

**Fig. 2:**
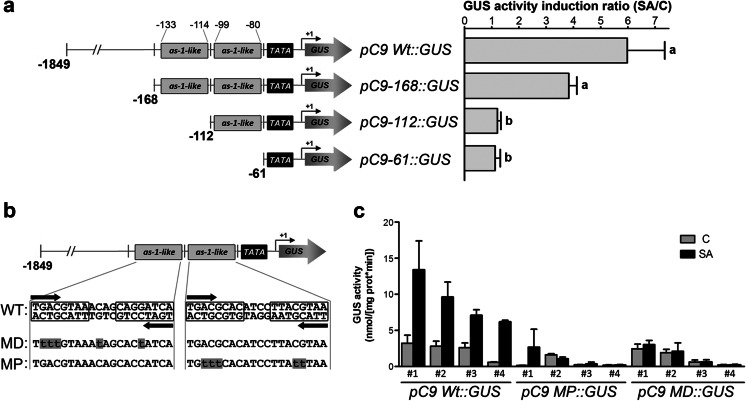
*GRXC9* promoter analysis by GUS reporter activity assays in *Arabidopsis* lines (**a**). The diagram represents the *GRXC9* promoter constructs used to generate the GUS reporter lines. The *numbers on the left side* indicate the size of the promoter region cloned to drive *GUS* expression, and the *numbers above the first construct* indicate the position from the transcriptional start site of the *as*-*1*-like elements in the *GRXC9* promoter. *pC9 WT*::*GUS* complete intergenic region for *GRXC9*, *pC9*-*168*::*GUS* promoter region containing the two *as*-*1*-like elements, *pC9*-*112*::*GUS* region containing the proximal *as*-*1*-like element, and *pC9*-*61*::*GUS* sequence of the promoter region containing the putative TATA box. To quantify GUS activity induced by SA in the reporter lines, 6 to 13 homozygous lines per construct were selected. Fifteen-day-old seedlings from each line were treated with SA 0.5 mM or 0.5× MS as a control (C) for 2.5 h. GUS activities were quantified in total protein extracts from each independent line and normalized with the total the amount of proteins (Online Resource 4). The ratio between SA treatment and its respective control was calculated, and the graph represents the average of GUS activity ratio obtained from the different lines. *Error bars* represent the standard error from three independent experiments. *Letters* indicate significant differences based on unpaired *t* test (*p* < 0.05). **b** Schematic representation of genetic constructs containing site-directed mutations in the *as*-*1*-like elements, in the context of the full *GRXC9* promoter sequence, and fused to *GUS*-coding region. *pC9 WT*::*GUS* (*WT* in the scheme) was used as a template to mutate the TGACG boxes (indicated by *black arrows*) from the two *as*-*1*-like elements (*highlighted in black boxes*). The mutated base pairs in distal *as*-*1*-like element (*MD*) and proximal *as*-*1*-like element (*MP*) are indicated in *lowercase* and highlighted in *gray boxes*. **c** Four independent homozygous GUS reporter lines carrying the *pC9 WT*::*GUS* and the mutated version in the proximal and distal *as*-*1*-like elements, *pC9 MP*::*GUS* and *pC9 MD*::*GUS*, respectively (described in **c**), were used to quantify GUS activity. Fifteen-day-old seedlings were treated with SA 0.5 mM (*black bars*) or 0.5× MS (*gray bars*) as control for 2.5 h, total proteins were prepared, and GUS activity was quantified and normalized with total protein concentration. The graph shows the mean value of three biological replicates for each line. *Error bars* represent the ±standard error. Data from a representative replicate is shown in Online Resource 5

In silico analysis of the *GRXC9* promoter sequence revealed the presence of several putative SA-responsive elements including two *as*-*1*-like elements, several W boxes, and isolated TGACG boxes (Online Resource 3). The proximal *as*-*1*-like element is located between −80 and −99 bp, and the distal one is located between −114 and −133 bp upstream of the transcriptional start site (Fig. 2a and Online Resource 3). In order to evaluate whether these elements mediate the SA-dependent transcriptional activation of the gene, we generated *Arabidopsis* transgenic lines harboring different versions of the *GRXC9* promoter fused to the *GUS* reporter gene. The constructs contained either the complete intergenic region (−1,849 to +26), considered as the full *GRXC9* promoter (*pC9 WT*::*GUS*), or truncated versions of this sequence that include the two *as*-*1*-like elements up to −168 bp (*pC9*-*168*::*GUS*), only the proximal *as*-*1*-like element up to −112 bp (*pC9*-*112*::*GUS*), or a minimal promoter up to −61 that includes the putative TATA box (*pC9*-*61*::*GUS*) (Fig. 2a and Online Resource 3).

We treated seedlings (from six to 13 independent homozygous lines for each construct) with 0.5× MS as control or with 0.5 mM SA for 2.5 h. We then quantified the basal and SA-induced GUS activities in total protein extracts (Online Resource 4). The responsiveness to SA of each construct was represented as the mean value of the GUS activity induction ratio (SA-induced/basal GUS activities) (Fig. 2a). An average of sixfold increase in GUS activity after SA treatment was recorded in lines that contain the full promoter (*pC9 WT*::*GUS*), which indicates that *GRXC9* gene expression is effectively activated by SA at the transcriptional level. Surprisingly, a very small promoter region up to −168 retains an important part of the SA responsiveness of the *GRXC9* full promoter (Fig. 2a and Online Resource 4). This region contains both *as*-*1*-like elements, the most proximal TGACG box and the most proximal W box, while lacking all the rest of the putative SA-responsive elements (Online Resource 3). In contrast, lines expressing the *pC9*-*112*::*GUS* and the *pC9*-*61*::*GUS* constructs were completely insensitive to SA treatment (Fig. 2a and Online Resource 4), suggesting that the loss of the distal *as*-*1*-like element, and/or the proximal W and TGACG boxes, is enough to abolish SA-responsiveness.

To further evaluate the importance of the two *as*-*1*-like elements in the SA-responsiveness of the promoter, we generated genetic constructs containing the full *GRXC9* promoter carrying point mutations in each *as*-*1*-like element (Fig. 2b). The nucleotides mutated in the *as*-*1-*like sequences were chosen considering the most conserved ones in the consensus *as*-*1*-like element detected in the cluster of genes induced by SA in a NPR1-independent manner (Blanco et al. 2009). Four independent *Arabidopsis* reporter lines were selected for each construct, either having mutations in the proximal (*pC9 MP*::*GUS*) or in the distal (*pC9 MD*::*GUS*) *as*-*1*-like elements (Fig. 2b). Seedlings from each line were treated with SA or 0.5× MS, and the GUS activity was quantified. As shown in Fig. 2c (see also Online Resource 5 for data from a representative replicate), lines that carry mutations in any of the two *as*-*1* elements no longer respond to SA treatment.

These results indicate that the increase in *GRXC9* transcript levels in response to SA is mainly due to transcriptional activation of the gene mediated by this hormone and that the loss of any of the *as*-*1*-like elements is enough to abolish this activation. Both elements are functional, essential, and sufficient for SA responsiveness of the *GRXC9* promoter.

### SA-Induced *GRXC9* Expression Is Abolished in *tga2*-*1*/*tga5*-*1*/*tga6*-*1* Triple Mutants, While TGA2 and TGA3 Are Constitutively Bound to the *GRXC9* Promoter In Vivo

**Fig. 3:**
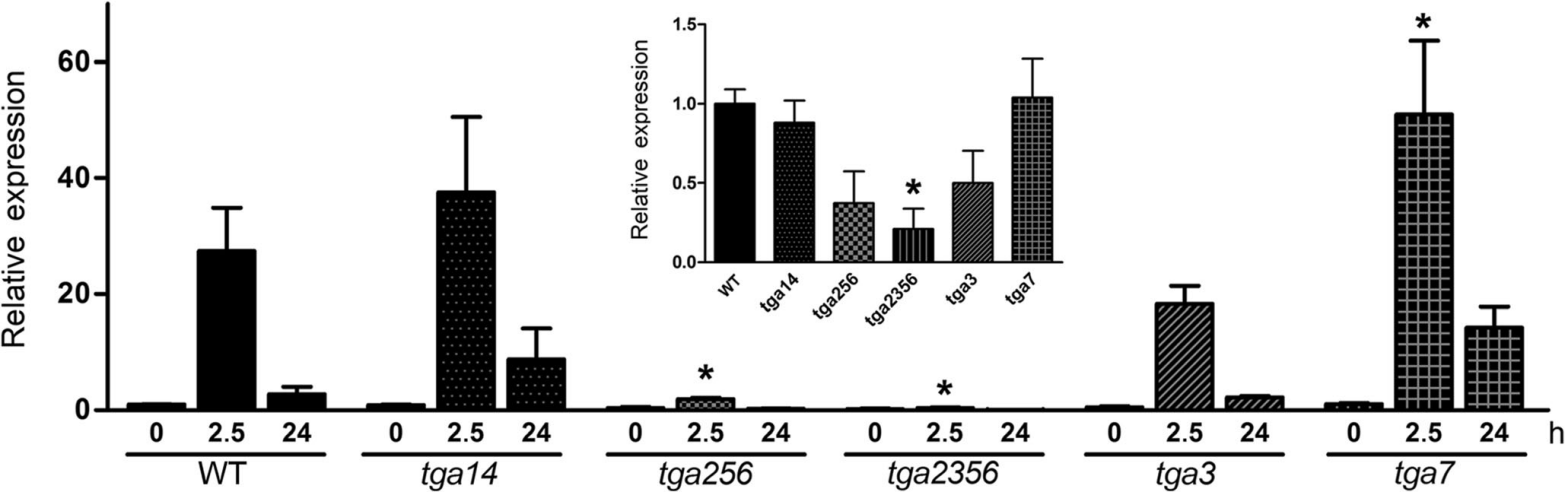
*GRXC9* gene expression in *tga* mutant backgrounds. Expression analysis of the *GRXC9* gene was evaluated by RT-qPCR in 15-day-old seedlings of WT, *tga1*-*1tga4*-*1* (*tga14*), *tga3*-*1* (*tga3*), *tga7*-*1* (*tga7*), *tga2*-*1tga5*-*1tga6*-*1* (*tga256*), and *tga2*-*1tga3*-*1tga5*-*1tga6*-*1* (*tga2356*) genotypes, under basal conditions (*insert*) and after treatment with SA 0.5 mM or 0.5× MS as a control for 2.5 and 24 h. The *GRXC9* relative expression was calculated by normalizing the expression level of *GRXC9* with the expression level of the housekeeping gene *Clathrin adaptor complex subunit* and with the WT basal condition. *Error bars* represent the ±standard error from three biological replicates (*p < 0.05, compared to the WT genotype in a two-way ANOVA and Bonferroni post test)

**Fig. 4:**
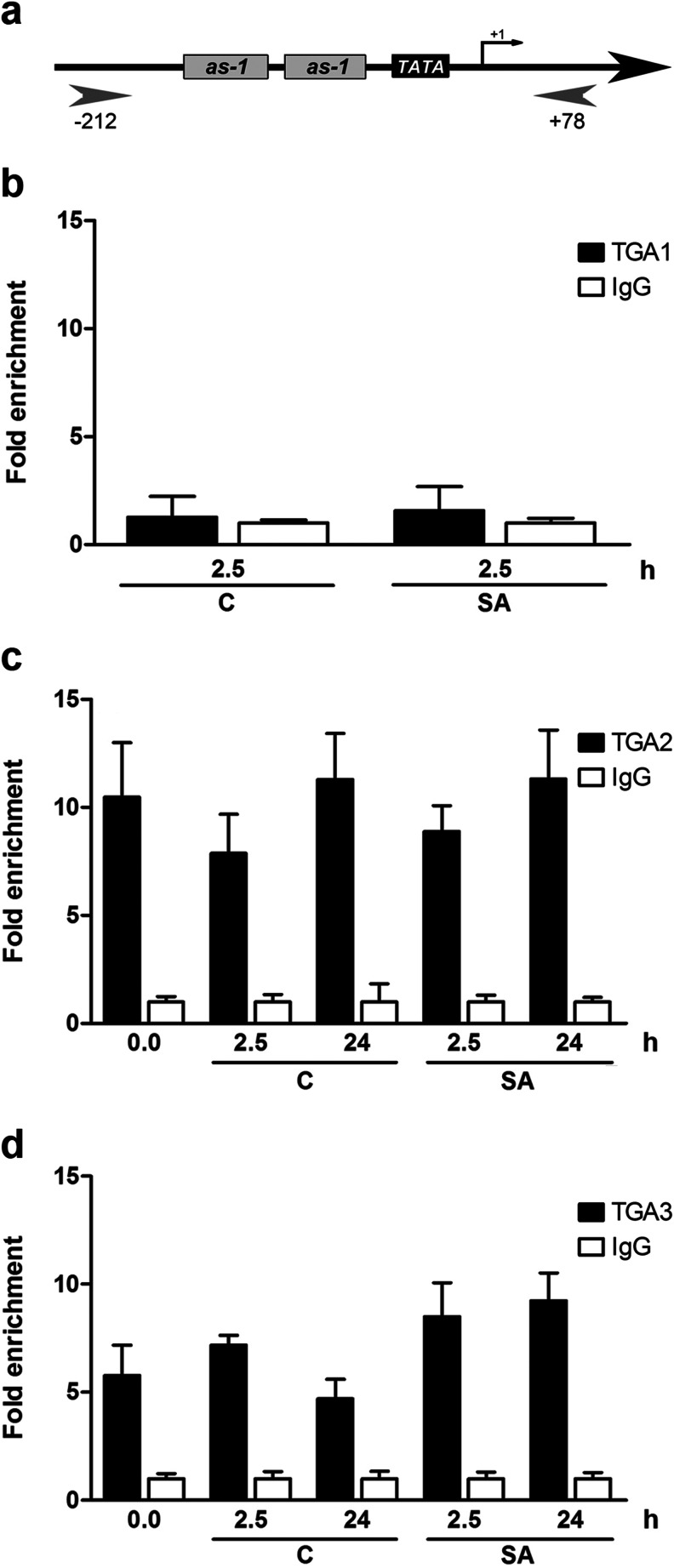
Analysis of TGAs binding to *as*-*1*-like elements of the *GRXC9* promoter by ChIP-qPCR assays. **a** Diagram of the *GRXC9* promoter region amplified in the ChIP-qPCR assays. The *arrowheads* indicate the location of the primers used to quantify the DNA of the *GRXC9* promoter bound to TGAs by qPCR. Fifteen-day-old plants treated with 0.5 mM SA (*SA*) or 0.5× MS as control (*C*) for 2.5 and 24 h were used to perform ChIP-qPCR assays. Antibodies raised against TGA1 (**b**), TGA2 (**c**), and TGA3 (**d**) transcription factors (in *black bars*) or IgG as a control (*white bars*) were used for the ChIP assays. qPCR analyses to quantify the DNA recovered from the ChIP were performed using the primers described in **a**. The values for the immunoprecipitated DNA samples are expressed as fold enrichment with the specific antibody over a nonspecific immunoprecipitation condition (IgG). *Error bars* represent ±standard error of three biological replicates

It has been reported that *GRXC9* induction by SA treatment is abolished in the TGA class II triple mutant (*tga2*-*1*/*tga5*-*1*/*tga6*-*1*) (Blanco et al. 2009; Ndamukong et al. 2007). Nevertheless, the possible participation of other members of the TGA family proteins, as well as the direct binding of TGA factors to the *GRXC9* promoter, has not been addressed. With this purpose in mind, we first analyzed the SA-induced expression of *GRXC9* by RT-qPCR in different *tga* mutant backgrounds (Fig. 3). Considering that TGAs belonging to class I (TGA1 and TGA4) and class II (TGA2, TGA5, and TGA6) show different degrees of redundancy (Zhang et al. 2003; Kesarwani et al. 2007), we used the double and triple mutant, respectively. The redundancy of TGA class III (TGA3 and TGA7) has not been demonstrated, and thus we analyzed the single mutants for each gene (Kesarwani et al. 2007). We correlated the expression data with in vivo binding assays of TGA factors to the *GRXC9* promoter after SA treatment using chromatin immunoprecipitation (ChIP) assays (Fig. 4). ChIP-qPCR assays were performed in WT plants treated with SA or 0.5× MS as control, using primers that amplify a 290-bp fragment (−212 to +78 region) that includes the basal promoter and the *as*-*1*-like elements (Fig. 4a). Antibodies that specifically recognize TGA1, TGA2, and TGA3, raised against the divergent N terminal regions, were used (Lam and Lam 1995).

In the double mutant of class I TGAs (*tga1*-*1*/*tga4*-*1*), *GRXC9* expression was early and transiently activated reaching its peak 2.5 h after SA treatment, as in WT plants (Fig. 3). Accordingly, the in vivo binding of TGA1 to the *GRXC9* promoter, evaluated by ChIP-qPCR, was not detected either under control conditions or after 2.5 h of SA treatment (Fig. 4b). These results indicate that class I TGAs are not involved in *GRXC9* induction.

In contrast, *GRXC9* induction by SA was significantly reduced in the triple mutant of TGA class II (*tga2*-*1*/*tga5*-*1*/*tga6*-*1*), compared to WT plants (Fig. 3), although a slight increase of the transcript after 2.5 h of SA was observed. On the other hand, the induction level by SA was 33.4 % reduced in the *tga3*-*1* mutant compared to WT plants, albeit this difference was not statistically significant (Fig. 3). To better evaluate the importance of TGA3 in *GRXC9* expression, we also assayed the quadruple *tga2*-*1*/*tga3*-*1*/*tga5*-*1*/*tga6*-*1* mutant background. Although the lack of TGA class II had a striking negative effect on SA-induced *GRXC9* transcription, the slight increase in messenger RNA (mRNA) levels after 2.5 h of SA treatment seen in the triple mutant is no longer observed in the quadruple mutant (Fig. 3). These results support that TGA class II, and to a lesser extent TGA3, is involved in *GRXC9* induction by SA. Interestingly, mutations in TGA class II and TGA3 factors also have an effect on the basal levels of *GRXC9* expression (Fig. 3, insert). Compared to WT plants, basal *GRXC9* transcript levels are reduced in the single *tga3*-*1* and in the triple *tga2*-*1*/*tga5*-*1*/*tga6*-*1* mutants, and this difference is higher and only statistically significant in the quadruple *tga2*-*1*/*tga3*-*1*/*tga5*-*1*/*tga6*-*1* mutant (Fig. 3 insert). Supporting these expression results, ChIP-qPCR assays show that TGA2 and TGA3 are constitutively bound to the *GRXC9* promoter, either in the presence or in the absence of SA stimulus (Fig. 4c, d).

Surprisingly, the lack of TGA7 produces a significant increase in *GRXC9* induction by SA (Fig. 3), suggesting that TGA7 can play a negative role in this control mechanism. We did not detect involvement of TGA factors in repressing *GRXC9* expression under basal conditions (Fig. 3, insert), in contrast to what was previously reported for *PR*-*1* expression (Kesarwani et al. 2007).

Together, these results indicate that constitutive binding of the TGA2 factor to the *GRXC9* promoter is essential for transcriptional activation mediated by SA. Even though TGA3 is also constitutively bound, its role is more important in the basal than in the SA-induced *GRXC9* expression.

Considering that the constitutive binding of TGA2 and TGA3 to the *GRXC9* promoter detected in vivo does not correlate with the constitutive expression of the gene, we propose that these TGA factors could bind either as homo or heterodimers, without being directly able to transactivate transcription. In order to evaluate the potential homodimerization and heterodimerization ability of TGA class II and TGA3 factors, as well as their transactivation activity, we performed yeast one and two hybrid assays. For this purpose, we cloned the CDS of TGA factors in frame with the DNA-binding domain (BD) or the activation domain (AD) of the yeast Gal4 factor. Interactions between TGA factors were evaluated in yeast by qualitative assays of the β-galactosidase reporter gene, whose expression is controlled by four copies of the Gal4-responsive element. The transactivation ability of the TGA factors was evaluated in assays using the TGA factors fused to the BD-Gal4 and the empty pDEST22 vector. Our results indicate that TGA2, TGA3, TGA5, and TGA6 cannot transactivate the β-galactosidase gene in a yeast system, reflected by the null activity of the reporter enzyme (Online Resource 6, first lane). Furthermore, the two-hybrid assays show that TGA class II and TGA3 factors are able to homodimerize and also heterodimerize among them (Online Resource 6). The interaction between NPR1 and TGA2 proteins was used as a positive control in these assays (Fan and Dong 2002).

These results support the idea that TGA2 and TGA3 bind to the *GRXC9* promoter, most probably through the SA-responsive *as*-*1*-like elements, both as homodimers or heterodimers, without acting directly in transactivation.

### SA Induces the Transient Recruitment of RNA Polymerase II to the *GRXC9* Promoter by a TGA Class II-Dependent Mechanism

**Fig. 5:**
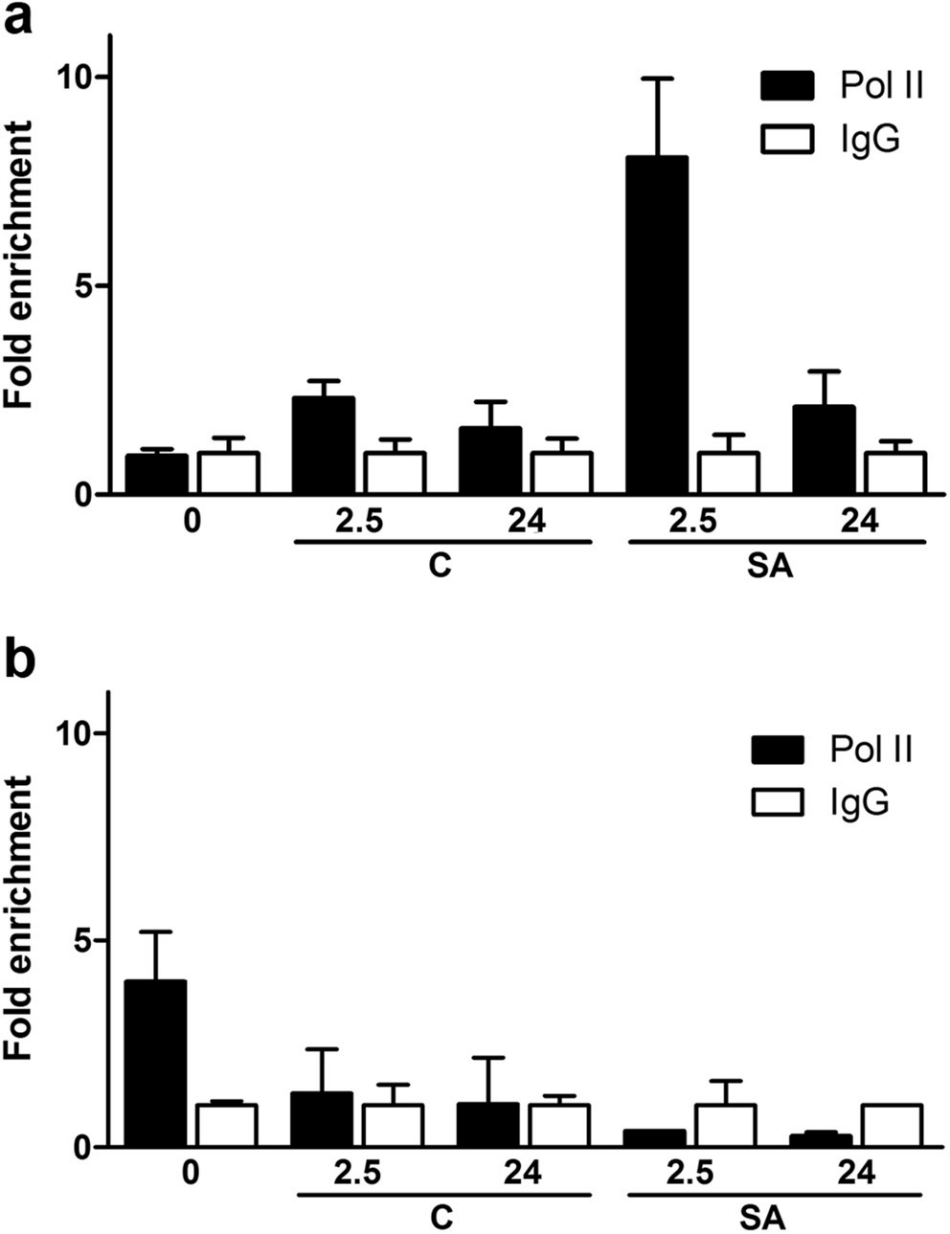
Recruitment of the RNA Pol II to the *GRXC9* promoter region by ChIP-qPCR assays. WT (**a**) and *tga2*-*1 tga 5*–*1 tga 6*–*1* (**b**) plants treated with 0.5 mM SA (*SA*) or 0.5× MS as control (*C*) for 0, 2.5, and 24 h were used to perform ChIP-qPCR assays. The ChIP assays were performed with a polyclonal antibody raised against the Pol II (*black bars*) or with a purified IgG (*white bars*) as a control. The qPCR analyses to quantify the DNA recovered from the ChIP were performed using the primers described in Fig. 4a. The values for the immunoprecipitated DNA samples were expressed as fold enrichment with the specific antibody over a nonspecific immunoprecipitation condition (IgG). *Error bars* represent ±standard error of three biological replicates

We evaluated whether, independently of the constitutive binding of TGA2 and TGA3 to the *GRXC9* promoter, the transient increase in *GRXC9* transcript levels correlates with a transient recruitment of Pol II to the *GRXC9* promoter, induced by SA. In order to test this, *Arabidopsis* seedlings were treated with 0.5 mM SA or 0.5× MS as a control, and ChIP assays were performed at different times. We used antibodies that recognize the N-terminal domain of Pol II from *Arabidopsis* and the set of primers previously described (Fig. 4a). Interestingly, we found a good correlation between the increment in *GRXC9* mRNA levels (Fig. 3, Online Resource 1a) and the recruitment of Pol II to the *GRXC9* promoter triggered by SA (Fig. 5a). Similarly, the recruitment of Pol II, as well as *GRXC9* expression, was abolished in the *tga2*-*1*/*tga5*-*1*/*tga6*-*1* triple mutant (Figs. 5b and 3).

Taken together, these results indicate that SA triggers the transient recruitment of Pol II to the basal *GRXC9* promoter, which explains the transient increase in *GRXC9* transcript levels triggered by SA. On the other hand, the fact that the increase in *GRXC9* transcript levels and the Pol II recruitment are impaired in the *tga2*-*1*/*tga5*-*1*/*tga6*-*1* triple mutant suggests that the constitutive binding of TGA factors is required for differential Pol II recruitment to the promoter.

### Overexpression of *GRXC9* Downregulates the Expression of its Endogenous Gene

It has been shown that TGA2 and *GRXC9* are able to interact in vivo and that this interaction may have a role in controlling the expression of genes involved in the defense response triggered by jasmonic acid (Ndamukong et al. 2007). However, the significance of this interaction in the SA response has not been addressed. Our results indicate that TGA2 is a key factor in the transcriptional induction of *GRXC9*, and thus we evaluated whether the overexpression of *GRXC9* has an effect in its own transient SA-dependent transcriptional induction.

**Fig. 6:**
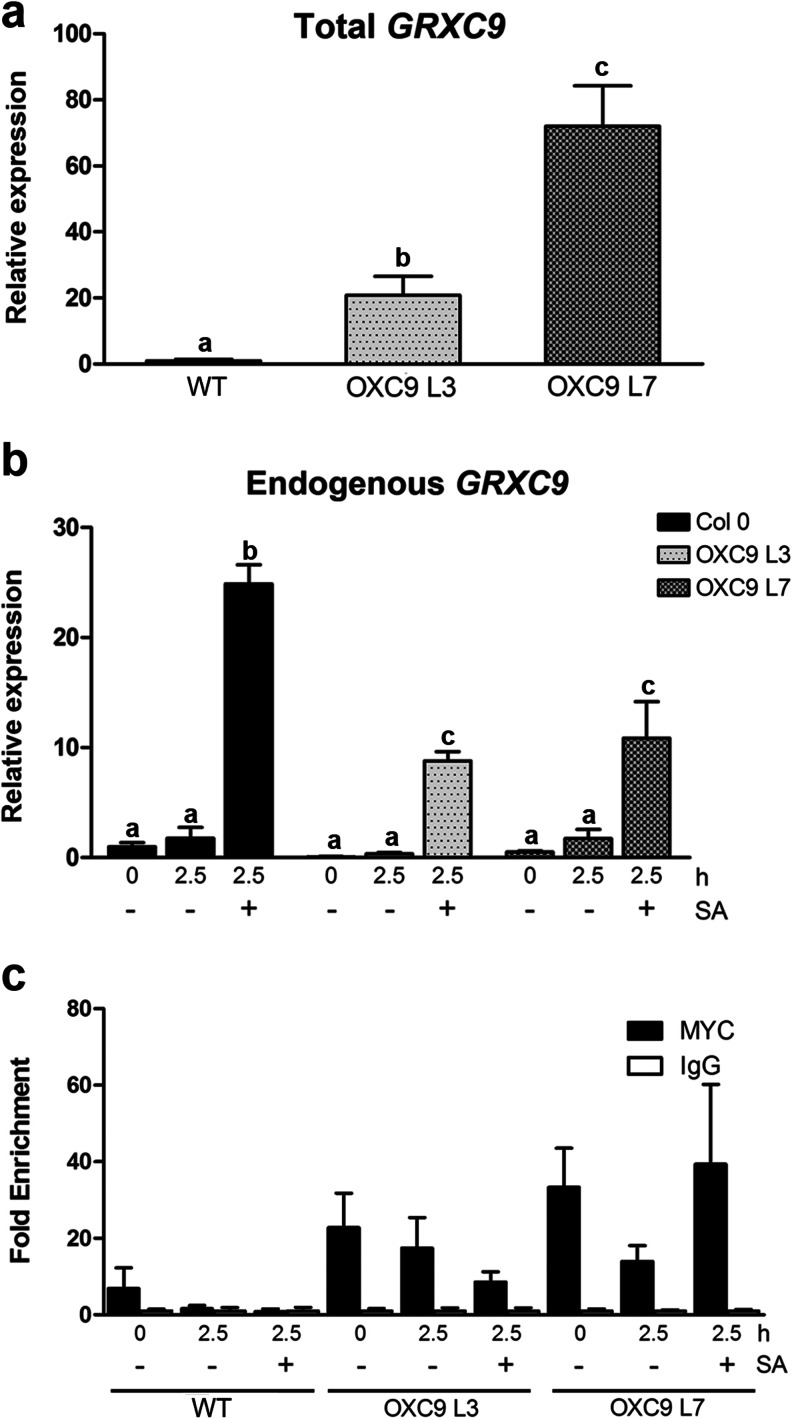
Effect of *GRXC9* overexpression on *GRXC9* gene expression and on binding to the *GRXC9* promoter. The levels of total (**a**) and endogenous (**b**) *GRXC9* transcripts were detected by RT-qPCR in 15-day-old seedlings of WT and *GRXC9* OE lines (L3 and L7) treated with SA 0.5 mM or 0.5× MS as a control for 2.5 h. *GRXC9* relative expression was calculated by normalizing the expression level of *GRXC9* with the expression level of the housekeeping gene *YLS8* and to the WT basal condition. *Error bars* represent the ±standard error. *Letters above the bars* indicate significant differences based on unpaired *t* test (*n* = 3, *p* < 0.05). The binding of *GRXC9*-Myc protein to the endogenous promoter of *GRXC9* was evaluated by ChIP-qPCR (**c**). WT plants and the two overexpressor lines (OXC9 L3 and OXC9 L7) were evaluated in the conditions mentioned above. The immunoprecipitation was performed with commercial antibodies raised against the MYC-tag (*black bars*) and a nonspecific IgG (*white bars*) as control. The qPCR analyses to quantify the DNA recovered from the ChIP were performed using the primers described in Fig. 4a. The values for the immunoprecipitated DNA samples were expressed as fold enrichment of the specific antibody over a nonspecific immunoprecipitation condition (IgG). *Error bars* represent ±standard error of three biological replicates

We produced WT transgenic lines harboring the CaMV 35S promoter controlling the expression of the *GRXC9*-coding region fused to the immunological cMyc tag. We chose two homozygous lines (L3 and L7) with high constitutive expression levels of *GRXC9* (Fig. 6a). We then evaluated the levels of the endogenous *GRXC9* transcript by RT-qPCR in WT and overexpressor lines treated with SA or 0.5× MS as control for 2.5 h. As shown in Fig. 6b, SA induction of the endogenous *GRXC9* gene was significantly reduced in both overexpressor lines to less than 50 % of the SA induction observed in WT plants. Although basal levels of *GRXC9* expression are reduced in both overexpressor lines compared to WT, these differences were not statistically significant (Fig. 6b). As a control, we show that overexpression of *GRXC9* does not affect basal or SA-induced *PR*-*1* gene expression (Online Resource 7). These results suggest that *GRXC9* negatively regulates the expression of its own gene.

To further investigate the role of *GRXC9* in the regulation of its own gene, we used ChIP-qPCR assays in the overexpressor lines to evaluate whether the *GRXC9*-Myc protein is associated with the TGA2/TGA3 complex bound to the *as*-*1*-like elements in the *GRXC9* promoter. Interestingly, we detected that *GRXC9*-Myc protein effectively forms part of the protein complex bound to the promoter (Fig. 6c), strongly suggesting that *GRXC9* regulates its own gene expression through binding to TGA factors while they are bound to the DNA.

## Discussion

In this study, we explore the mechanism of control of gene expression via an SA-dependent and NPR1-independent pathway in *Arabidopsis*, using *GRXC9* as a model gene. First, we showed evidence for the induction of *GRXC9* expression through this pathway in response to UVB stress, validating its activation in the context of a defense response to stress (Fig. 1a). By assaying *in planta GRXC9*-GUS reporter activity (Fig. 2a) and in vivo binding of Pol II to the *GRXC9* promoter (Fig. 5a), we showed that the control of *GRXC9* gene expression by SA is exerted at the initiation of transcription. Accordingly, we established that TGA class II factors (Figs. 3 and 5b), as well as the two *as*-*1*-like elements located in the *GRXC9* proximal promoter (Fig. 2c), are essential for the induction of *GRXC9* expression by SA. The constitutive binding of TGA2 and TGA3 factors to the *GRXC9* promoter, as detected by in vivo ChIP assays (Fig. 4), indicates that the inducing effect of SA is not due to an increase in binding of TGA factors to the *as*-*1*-like elements. TGA class II and TGA3 factors have the capacity to interact with each other, as detected in yeast by two-hybrid assays (Online Resource 6), suggesting that TGA2 and TGA3 can bind to the *GRXC9* promoter as homodimers or heterodimers. Furthermore, TGA class II and TGA3 factors did not show transactivation capacity in yeast one-hybrid assays (Online Resource 6). Therefore, even though TGA class II factors are essential for recruiting Pol II to the *GRXC9* promoter (Fig. 5b), additional coregulators are required for transactivation. Finally, we showed that overexpressed *GRXC9*-Myc protein binds to the TGA-containing complex at the *GRXC9* promoter and inhibits SA-mediated induction of the gene (Fig. 6). This result, together with previous evidence showing that the *GRXC9* protein can interact with TGA2 in the nucleus (Ndamukong et al. 2007), suggests that *GRXC9* negatively controls its own gene through binding to TGA factors while they are bound to the DNA, turning off gene expression.

### Role of the SA-Dependent and NPR1-Independent Pathway in the Stress Defense Response

The existence of an SA-dependent and NPR1-independent pathway for the expression of genes with a putative antioxidant and/or detoxifying roles in defense, such as *GRXs*, *GSTs*, and *UGTs* (coding for UDP-glucosyl transferases), has been reported by our group and by others (Lieberherr et al. 2003; Blanco et al. 2005, 2009; Uquillas et al. 2004; Langlois-Meurinne et al. 2005; Fode et al. 2008). The induction of two *GST* (Lieberherr et al. 2003) and three *UGT* genes (Langlois-Meurinne et al. 2005), through an SA-dependent and NPR1-independent mechanism, was reported in *Arabidopsis* plants inoculated with avirulent strains of *Pseudomonas syringae* pv. *tomato* (*Pst*). Here, we validate the activation of the *GRXC9* gene by this pathway under an abiotic stress condition such as UVB radiation that, like the immune reaction induced by avirulent *Pst* strains, triggers an SA-mediated defense response (Surplus et al. 1998).

On the other hand, members of *GST* and *UGT* gene families have also been found to be responsive to treatments with oxilipins (including JA) and xenobiotic chemicals like 2,4-D, in the context of the chemical detoxification process. Based on the overlap of some *GST* and *UGT* (early NPR1-independent SAIG (Blanco et al. 2009) that are also responsive to oxilipins/xenobiotics (Fode et al. 2008; Baerson et al. 2005; Mueller et al. 2008), as well as on the involvement of common TGA factors and *as*-*1*-like elements in their transcriptional control (as discussed in the next section), it was assumed that exogenous treatments with SA unspecifically induced the chemical detoxification process (Fode et al. 2008; Gatz 2012). Results shown in this work for *GRXC9* and in other works for the *GSTF2*, *GSTF6*, *UGT73B3*, *UGT73B5*, and *UGT73D1* genes (Lieberherr et al. 2003; Langlois-Meurinne et al. 2005) clearly argue against this idea, indicating that these antioxidant/detoxifying genes are activated by an endogenous stress-driven and SA-mediated pathway, which is distinct from the NPR1-dependent pathway that activates defense genes such as *PR*-*1*. The rapid and transient expression of genes with antioxidant and detoxifying roles could be important to restrict the oxidative burst produced in the infected/damaged tissues, avoiding the oxidative damage of systemic tissues.

### Mechanistic Aspects of the Transcriptional Control of *GRXC9* Expression via an SA-Dependent and NPR1-Independent Pathway. Involvement of TGA Class II Factors and *as*-*1*-Like Promoter Elements

Evidence provided in this paper further supports the idea that, even though *as*-*1*-like and isolated TGACG boxes bind the same class of TGA factors, they are functionally different. These factors respond to different pathways to control the expression of distinct groups of genes that are activated at different times during the defense response, using different mechanism for promoter recognition and activation.

In *Arabidopsis*, *as*-*1*-like elements are overrepresented in promoters of genes that code for enzymes with antioxidant or detoxifying activity that is responsive to exogenous application of SA, xenobiotics, and oxilipins (Blanco et al. 2009; Fode et al. 2008; Köster et al. 2012). Functional requirement for *as*-*1*-like elements has been previously reported for only a couple of these *Arabidopsis* genes: *GST6* coding for a *GST* inducible by xenobiotics, H_2_O_2_, and SA (Chen and Singh 1999) and *CYP81D11* coding for a cytochrome P450 inducible by xenobiotics (Köster et al. 2012). The functional analysis of the *as*-*1*-like elements from the *GRXC9* promoter described in this work represents the first functional promoter analysis performed in an early SA-dependent and NPR1-independent gene activated under an abiotic stress.

The *GRXC9* gene has two contiguous *as*-*1*-like elements in its promoter sequence (Fig. 2b and Online Resource 3). In both cases, the second TGACG box is less conserved than the first one, as previously described for other *as*-*1*-like elements (Ellis et al. 1993). The functional analysis of the *GRXC9* gene promoter clearly indicates that both *as*-*1*-like elements are essential for SA-mediated expression of the gene (Fig. 2). Interestingly, the *all or none* effect of mutating any *as*-*1*-like element, instead of additive or synergistic effects, indicates that both elements must work together in the formation of transcriptional complexes.

In contrast, isolated TGACG boxes are enriched in promoters of SAIGs by an NPR1-dependent pathway (Maleck et al. 2000). Functional requirement of a TGACG box for SA-mediated expression was demonstrated for *PR*-*1* and *NIMIN1* promoters, which are known to be induced by SA via an NPR1-dependent mechanism (Lebel et al. 1998; Fonseca et al. 2010). In the case of the *PR*-*1* gene, isolated TGACG boxes control its transcriptional activation by SA and its repression under basal conditions (Lebel et al. 1998).

Interestingly, this work shows that common TGA factors (TGA class II and TGA3) recognize *as*-*1*-like elements and TGACG boxes, being therefore involved in different transcriptional control processes. In *Arabidopsis*, TGA class II factors have been reported to be essential in several processes: the basal repression and the SA-mediated and NPR1-dependent induction of plant TGACG box-containing genes, such as *PR1* gene (Rochon et al. 2006; Kesarwani et al. 2007; Zhang et al. 2003), the induction of *as*-*1*-like-containing plant genes belonging to the chemical detoxification process, in response to treatments with xenobiotics and oxilipins (including JA) (Fode et al. 2008; Mueller et al. 2008; Stotz et al. 2013), the induction of JA/ethylene-inducible genes like *PDF1.2* and its negative modulation by SA (Ndamukong et al. 2007; Zander et al. 2010), and the SA-mediated induction of early and NPR1-independent SAIGs (Blanco et al. 2009), the last one supported by the results of this work.

One interesting conclusion of this work is that TGA2 is involved in different signaling pathways that operate at different times in the response to stress (UVB in this case). One of these pathways leads to the expression of early SA-dependent and NPR1-independent genes, such as *GRXC9*; the other pathway leads to the expression of late SA- and NPR1-dependent genes, such as *PR1* (Fig. 1). Furthermore, in the case of *GRXC9*, TGA2 is bound to the promoter during all the phases of its expression profile (Fig. 4), being essential for the transient increase in transcript levels (Fig. 3) and recruitment of the Pol II (Fig. 5). The question is that how is the activity of TGA factors that are involved in different mechanisms of transcriptional control and that act at different times after stress controlled?

**Fig. 7:**
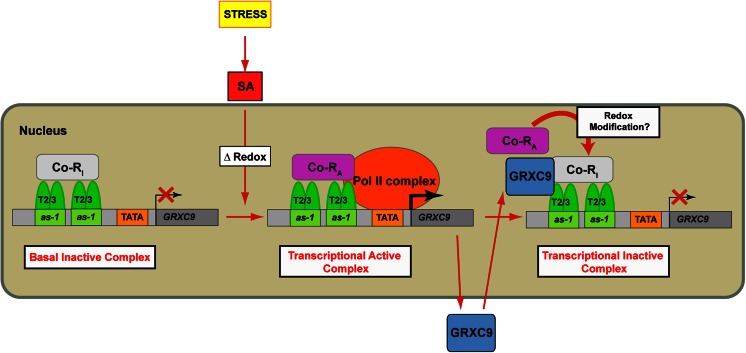
Mechanistic model for the transcriptional control of *GRXC9* expression by stress, via an SA-dependent and NPR1-independent pathway in *Arabidopsis*. Homodimers or heterodimers of TGA2 and TGA3 (T2/3) are constitutively bound to the two *as*-*1*-like elements of the *GRXC9* promoter, acting as a platform for the formation of transcriptionally inactive and active complexes. Under basal conditions, an inactive form of a coregulator (Co-R_I_) is bound to the TGA2-3/*as*-*1*-like complex forming a basal complex that impairs recruitment of the Pol II to the *GRXC9* promoter. Upon stress, SA is rapidly accumulated promoting the activation of the coregulator complex (switch from Co-R_I_ to Co-R_A_) that binds to the TGA2-3/*as*-*1*-like complex, allowing the formation of a transcriptionally active complex that recruits the Pol II basal machinery (Pol II complex) to the *GRXC9* basal promoter. Transcription of *GRXC9* leads to the accumulation of the *GRXC9* protein in the nucleus where it binds to the TGA2-3/*as*-*1*-like complex producing the inactivation of the coregulator complex (switch from Co-R_A_ to Co-R_I_) and therefore turning off *GRXC9* transcription. We speculate that the switch from Co-R_I_ to Co-R_A_ promoted by SA is produced by the oxidative modification of one of the proteins involved in the promoter complex, while the switch from Co-R_A_ to Co-R_I_ is produced by the protein’s reduction catalyzed by *GRXC9*

Results showed in this work prompt us to propose a model (Fig. 7) to explain how SA controls the transcription process of *GRXC9* in the context of the defense response to stress. According to our model, TGA2 and TGA3 (forming homodimers or heterodimers, T2/3) are constitutively bound to the two *as*-*1*-like elements of the *GRXC9* promoter. Under basal conditions, we propose that an inactive form of a coregulator complex (Co-R_I_) is bound to the TGA2-3/*as*-*1*-like complex, forming a transcriptionally inactive complex. Upon a stress condition, SA levels increase producing the activation of the coregulator complex (switch from Co-R_I_ to Co-R_A_). According to our results, Co-R_A_ must provide the transactivation activity for recruitment of the Pol II basal machinery (Pol II complex) to initiate transcription. According to our results with the *GRXC9* overexpressor lines (Fig. 6), we propose that the *GRXC9* protein is involved in turning off the SA-mediated activation of its own gene through a direct interaction with the TGA2-3/*as*-*1*-like complex. So, once *GRXC9* gene expression is induced, the *GRXC9* protein produced and translocated to the nucleus could bind the TGA2-3/*as*-*1*-like complex, as indicated by Ndamukong et al. (2007). We propose that *GRXC9* bound to the complex promotes the inactivation of the coregulator complex (Co-R_A_ to Co-R_I_), switching from a transcriptionally active to a transcriptionally inactive complex. *GRXC9* expression is turned off because Co-R_I_ does not have the ability to recruit Pol II basal machinery. This mechanism would allow a rapid transcriptional response to stress signals, through transient changes in the activity of a coregulator complex bound to the TGA2-TGA3/*as*-*1*-like platform complex preformed at the *GRXC9* promoter.

Results indicating that *GRXC9* and TGA2 interact in the nucleus and that *GRXC9* overexpression reduces the expression of the 2,4-D-inducible CaMV*as*-*1*::GUS transgene and the JA-inducible *PDF1.2* gene (Ndamukong et al. 2007) support the idea that *GRXC9* could play a more general role in controlling the expression of TGA class II-target genes. Interestingly, the binding of other CC-type *GRX* (ROXY1 and ROXY2) to TGA9/10 factors and their role in anthers development (Murmu et al. 2010) supports a role for CC-type *GRXs* in the control of gene expression. Considering that ROXY1 must be expressed in the nucleus to complement *roxy1* mutant and that *GRXC9* can also complement the *roxy1* mutant (Li et al. 2009), it can be inferred that nuclear *GRXC9* is required to exert its activity. Our results indicating that *GRXC9* binds to the TGA2-3/*as*-*1*-like complex support this idea.

One of the key questions raised by this model is what is the nature of the proteins that form the coregulator complex, either in its active (Co-R_A_) or inactive (Co-R_I_) forms. The existence of a corepressor complex that binds the TGA2-3/*as*-*1*-like complex under basal conditions (Fig. 7) was previously proposed for the 35SCaMV *as*-*1* in tobacco (Johnson et al. 2001; Butterbrodt et al. 2006). In the case of *GRXC9*, previous evidence showing that treatment with the protein inhibitor cycloheximide highly increases the basal and SA-induced levels of *GRXC9* transcripts suggests the existence of a repressor with a high turnover rate (Blanco et al. 2009). More recently, a subunit of the mediator complex (MED18) was found to be essential for suppression of *GRXC9* as well as *GRXS13* and *TRX*-*h5* expression in the absence of stress, suggesting that this subunit forms part of the Co-R_I_ complex (Lai et al. 2014). On the other hand, we show here evidence that although TGA2 and TGA3 are basally bound to the *GRXC9* promoter, their binding is not required for turning off the gene in the absence of stress. In fact, knockout mutants for TGA class II and TGA3 factors do not show increased basal levels of *GRXC9* transcripts (Fig. 3). Together, this evidence indicates that even though TGA2/TGA3 and Co-R_I_ (probably containing MED18) form part of the basal inactive complex, the presence of MED18 but not of TGA2/TGA3 factors is essential to repress *GRXC9* transcription.

Concerning proteins with coactivator function that could be part of the Co-R_A_ complex, we discard NPR1 and SCL14 proteins. In fact, the induction of *GRXC9* by SA is not only independent of the NPR1 protein ((Blanco et al. 2009) and Online Resource 1) but also independent of the SCL14 protein (Fode et al. 2008), which was previously identified as an interactor of TGA2 factor essential for the expression of a group of *as*-*1*-like-containing genes. Previously, a Dof protein named OBP1 was found to interact with TGA4 and TGA5 to enhance binding to the *as*-*1* element (Zhang et al. 1995); whether this kind of protein forms part of the Co-R_A_ complex remains to be elucidated. Therefore, further efforts are required for the identification of the protein(s) that form part of the Co-R_A_ complex that binds to the TGA2-3/*as*-*1*-like complex in *GRXC9* promoter.

Another interesting question raised by this model is what is the mechanism by which SA promotes the activation of the coregulator complex (switch from Co-R_I_ to Co-R_A_), as well as how *GRXC9* can promote the inactivation of the coregulator complex (switch from Co-R_A_ to Co-R_I_). We speculate in our model that a redox change promoted by SA accumulation can be responsible for the activation of the coregulator complex. This idea is supported by evidence indicating that SA promotes a biphasic change in the GSH/GSSG ratio, first an oxidative phase characterized by a decrease in GSH/GSSG ratio and then a reductive phase characterized by increase in GSH/GSSG ratio (Mou et al. 2003; Mateo et al. 2006). With respect to the mechanism of inactivation of the coregulator complex, we speculate that *GRXC9*, through its oxidoreductase activity, can catalyze the reduction of a protein that forms part of the coregulator complex, producing its inactivation. *GRXC9* could be a key piece in the redox control of the expression of genes controlled by TGA class II factors.

## Methods

### Plant Growth Conditions and Treatments

*Arabidopsis thaliana* wild-type (WT), *npr1*-*1* (Cao et al. 1994), *tga1*-*1*/*tga4*-*1*, *tga3*-*1*, *tga2*-*1*/*tga3*-*1*/*tga5*-*1*/*tga6*-*1* (Kesarwani et al. 2007) *tga*-*7*-*1*, *tga2*-*1*/*tga5*-*1*/*tga6*-*1* (Zhang et al. 2003), and *sid2*-*2* (Wildermuth et al. 2001) plants were in Columbia (Col-0) background. Seedlings were grown in vitro in 0.5× MS medium supplemented with 10 g/l sucrose and 2.6 g/l Phytagel (Sigma) under controlled conditions (16 h light, 80 μmol/m^2^/s, 22 ± 2 °C). For ChIP and gene expression assays, 15-day-old seedlings were floated on 0.5 mM SA (treatment) or 0.5× MS medium as a control and incubated for the indicated periods of time under continuous light (80 μmol/m^2^/s). For gene expression assays, whole seedlings were immediately frozen in liquid nitrogen and stored at −70 °C until RNA isolation. For ChIP assays, whole seedlings were processed immediately as described below. For UVB irradiation assays, 15-day-old seedlings were exposed to UVB light (0.07 mW/cm^2^) in a chamber equipped with two USHIO UVB F8T5.UB-V, UVP 3400401 fluorescent tubes (*λ* = 306 nm). As a control, we used nonirradiated seedlings.

### Genetic Constructs and Plant Transformation

Genetic constructs were generated using the Gateway technology following the manufacturer’s instructions (Invitrogen). The *GRXC9* promoter regions including the 5′UTR, −1,849 to +26; −168 to +26; −112 to +26; and −61 to +26, called in the text as pC9 WT, pC9-168, pC9-112, and pC9-61, respectively, were obtained by amplification from genomic DNA, using the oligonucleotides indicated in Online Resource 8. PCR fragments were cloned into the pENTR/SD/D-TOPO vector and then recombined into the pKGWFS7 vector to generate transcriptional fusions with *eGFP* and β-glucuronidase *GUS* reporter genes (Karimi et al. 2002). Site-directed mutation of the distal and the proximal *as*-*1*-like element was performed on the pC9 WT promoter fragment cloned into the pENTR/SD/TOPO vector, as previously described (Weiner et al. 1994). The site-directed mutations generated are shown in Fig. 2c, and the oligonucleotides used to produce these mutations are listed in the Online Resource 8. The purified PCR products were recombined into the pKGWFS7 vector. In order to generate *GRXC9* overexpressor lines, the *GRXC9*-coding region was amplified from complementary DNA (cDNA) using the primers described in the Online Resource 8. The PCR product was cloned into the pENTR/SD/D-TOPO vector and then recombined into the pBADcMyc vector to express the *GRXC9* protein fused to a c-Myc tag controlled by the 35S CaMV promoter. Final constructs were verified by sequencing and introduced into the *Agrobacterium tumefaciens* C58 strain. *Arabidopsis* plants were transformed by floral dip method. Transgenic seeds were selected in 0.5× MS solid medium supplemented with 50 μg/ml kanamycin for the GUS reporter lines or 15 μg/ml glufosinate-ammonium for the overexpressor lines. Stable homozygous transgenic lines were used for further analyses.

### GUS Assays

GUS activity was determined in control- and SA-treated seedlings from each transgenic line carrying the *GRXC9* promoter-driven GUS constructs described above. The 4-methylumbelliferyl-d-glucuronide was used as substrate, and the fluorescent product 4-methylumbelliferone was quantified, as previously described (Jefferson et al. 1987). Treatments were done by triplicate for each line, and the measurements were normalized with total protein content quantified using the Bradford assay (Bio-Rad).

### ChIP Assays

ChIP assays were performed as described (Saleh et al. 2008). Five microliters of the following antibodies were used for immunoprecipitation assays: Pol II polyclonal antibody (sc-33754, Santa Cruz Biotechnology), TGA1, TGA2, and TGA3 polyclonal antibodies (Lam and Lam 1995), c-Myc polyclonal antibody (A-14, sc-789, Santa Cruz), and normal purified IgG (A2609, Santa Cruz Biotechnology) used as control of a nonspecific antibody. The concentration of DNA in each sample (input chromatin and chromatin immunoprecipitated with either specific or nonspecific antibodies) was quantified by qPCR, using the Stratagene MX3000P® equipment and the Sensimix Plus SYBR Green Reagents (Quantece). Primers used to amplify the *GRXC9* promoter region containing the *as*-*1*-like elements (−212 to +78) are listed in Online Resource 8.

### Gene Expression Analysis

Total RNA was obtained from frozen samples using the TRIzol® Reagent (Invitrogen) according to the manufacturer’s instructions. cDNA was synthesized from each sample (2 μg of total RNA) with an ImProm II Kit (Promega). qPCR was performed with the Stratagene MX3000P® equipment. The expression levels of *GRXC9* and *PR*-*1* were calculated relative to the *YLS8* (AT5G08290) or *Clathrin adaptor complex subunit* (AT4G24550) genes. Primers used for each gene are listed in Online Resource 8.

### Yeast Two-Hybrid Assays

The coding regions of TGA factors (TGA2, TGA5, and TGA6 and TGA3) were cloned into the pDONR201 vector (Jakoby et al. 2002). These coding regions were then recombined into the pDEST22 vector, to produce a fusion protein with the Gal4 DNA-binding domain and into the pDEST32 vector, to produce a fusion protein with the transactivation domain of the Gal4 factor. Different combinations of two constructs were used to transform the SFY526 yeast strain (harboring the *Gal4RE*::*β*-*Gal* reporter construct), and qualitative assays for β-Gal activity were performed as described (Gietz and Schiestl 2007). Interaction between NPR1 and TGA2 was assayed as a positive control, and a combination of the pDEST32 and pDEST22 empty vectors was used as a negative control.

## Electronic supplementary material

Below is the link to the electronic supplementary material.

Online Resource 1 (GIF 141 kb)

High Resolution Image (TIFF 19063 kb)

Online Resource 2 (GIF 124 kb)

High Resolution Image (TIFF 20513 kb)

Online Resource 3 (GIF 139 kb)

High Resolution Image (TIFF 61163 kb)

Online Resource 4 (GIF 116 kb)

High Resolution Image (TIFF 14914 kb)

Online Resource 5 (GIF 119 kb)

High Resolution Image (TIFF 20470 kb)

Online Resource 6 (GIF 86 kb)

High Resolution Image (TIFF 57136 kb)

Online Resource 7 (GIF 126 kb)

High Resolution Image (TIFF 16613 kb)

Online Resource 8 (DOCX 21 kb)
