# Supplementary figures and images for: Transcriptional Control of Glutaredoxin GRXC9 Expression by a Salicylic Acid-Dependent and NPR1-Independent Pathway in Arabidopsis
Source: Plant Mol Biol Report. 2014 Aug 14;33(3):624–37. doi: 10.1007/s11105-014-0782-5 (PMC4677692; doi:10.1007/s11105-014-0782-5)

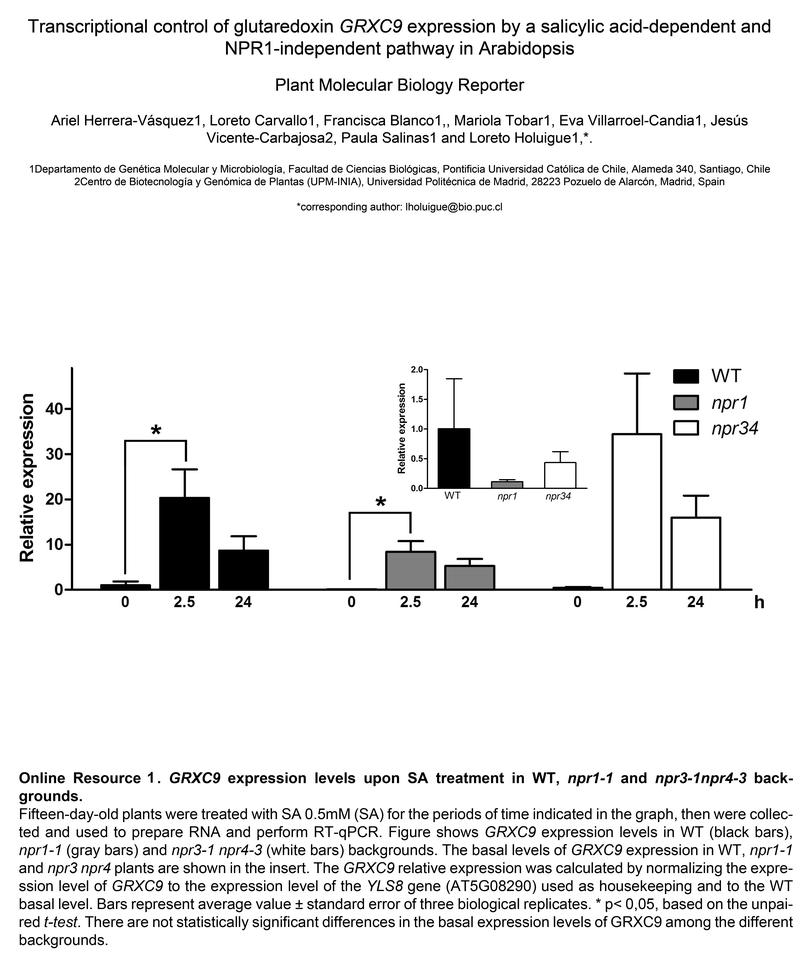

Supplement: Supplementary file 1 — (GIF 141 kb) [file 11105_2014_782_Fig8_ESM.gif]

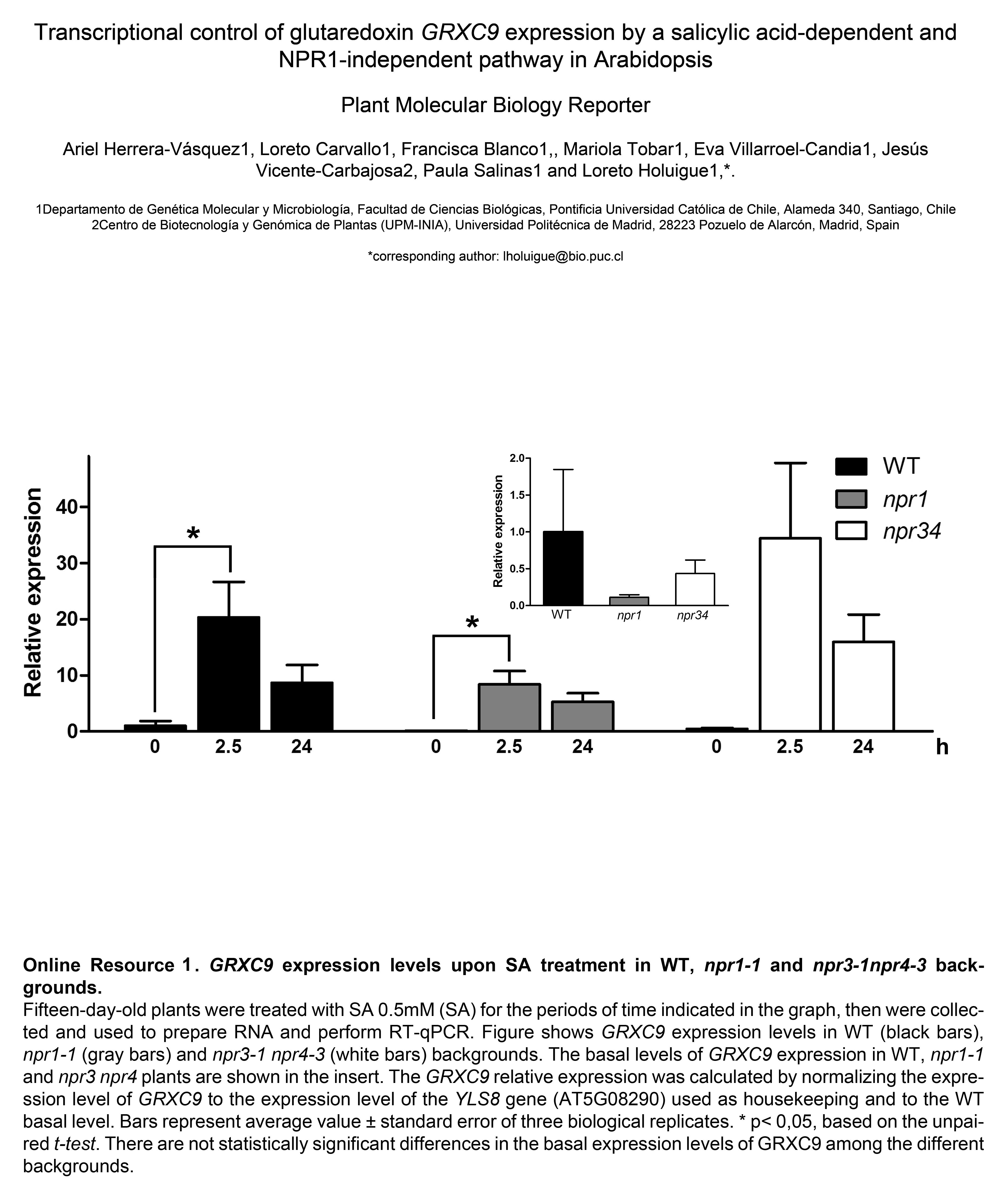

Supplement: Supplementary file 2 — High Resolution Image (TIFF 19063 kb) [file 11105_2014_782_MOESM1_ESM.tif]

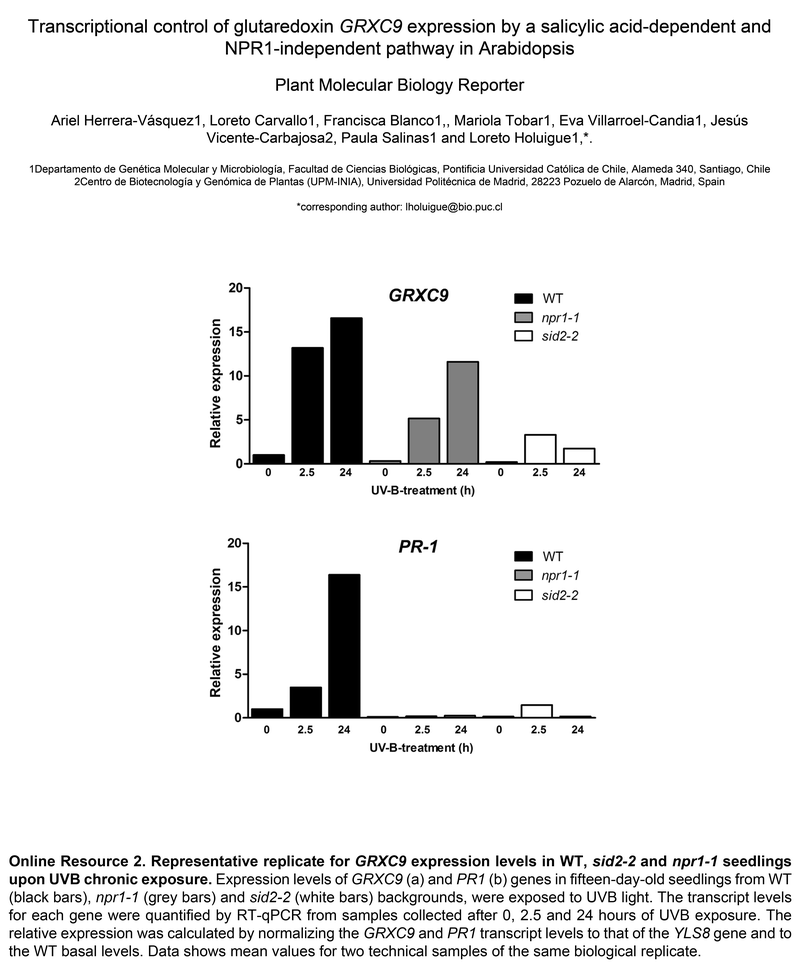

Supplement: Supplementary file 3 — (GIF 124 kb) [file 11105_2014_782_Fig9_ESM.gif]

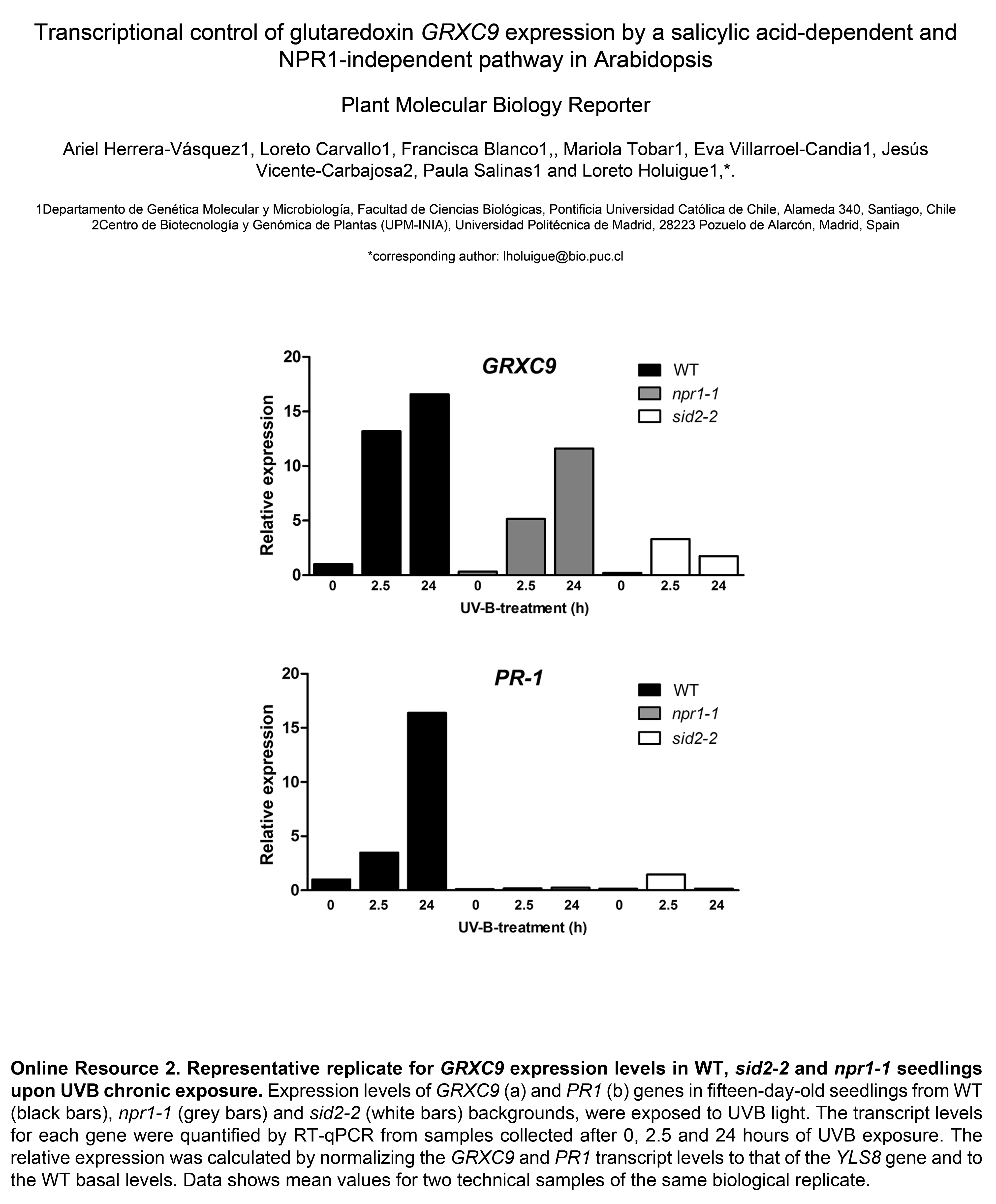

Supplement: Supplementary file 4 — High Resolution Image (TIFF 20513 kb) [file 11105_2014_782_MOESM2_ESM.tif]

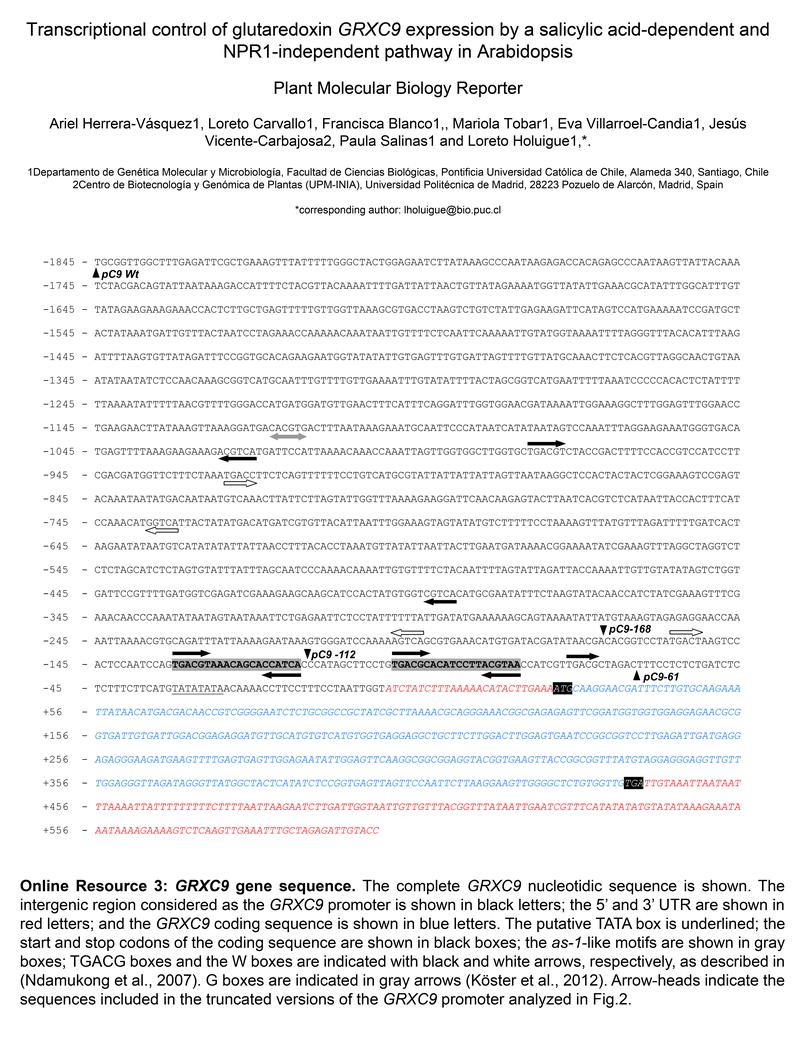

Supplement: Supplementary file 5 — (GIF 139 kb) [file 11105_2014_782_Fig10_ESM.gif]

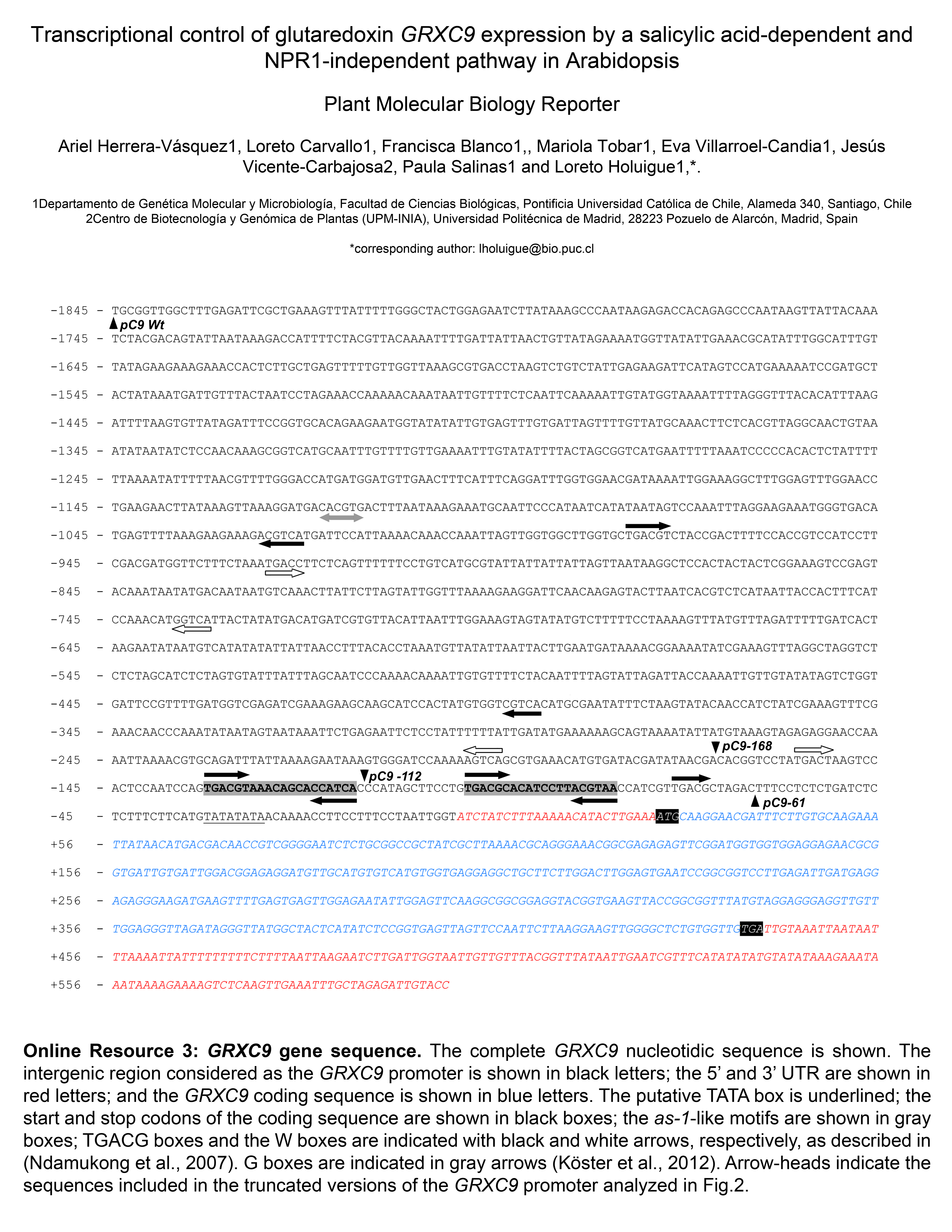

Supplement: Supplementary file 6 — High Resolution Image (TIFF 61163 kb) [file 11105_2014_782_MOESM3_ESM.tif]

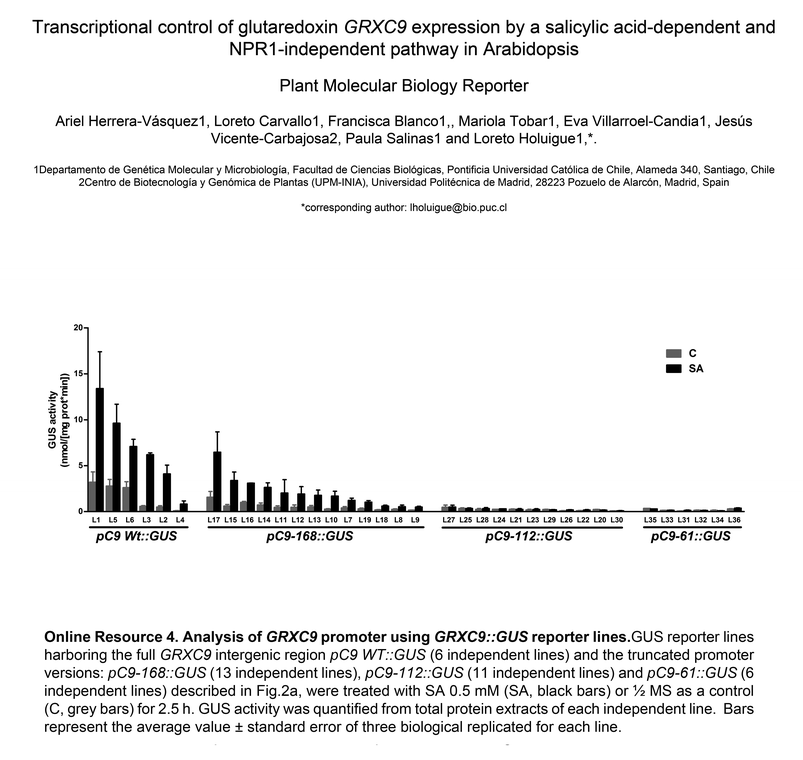

Supplement: Supplementary file 7 — (GIF 116 kb) [file 11105_2014_782_Fig11_ESM.gif]

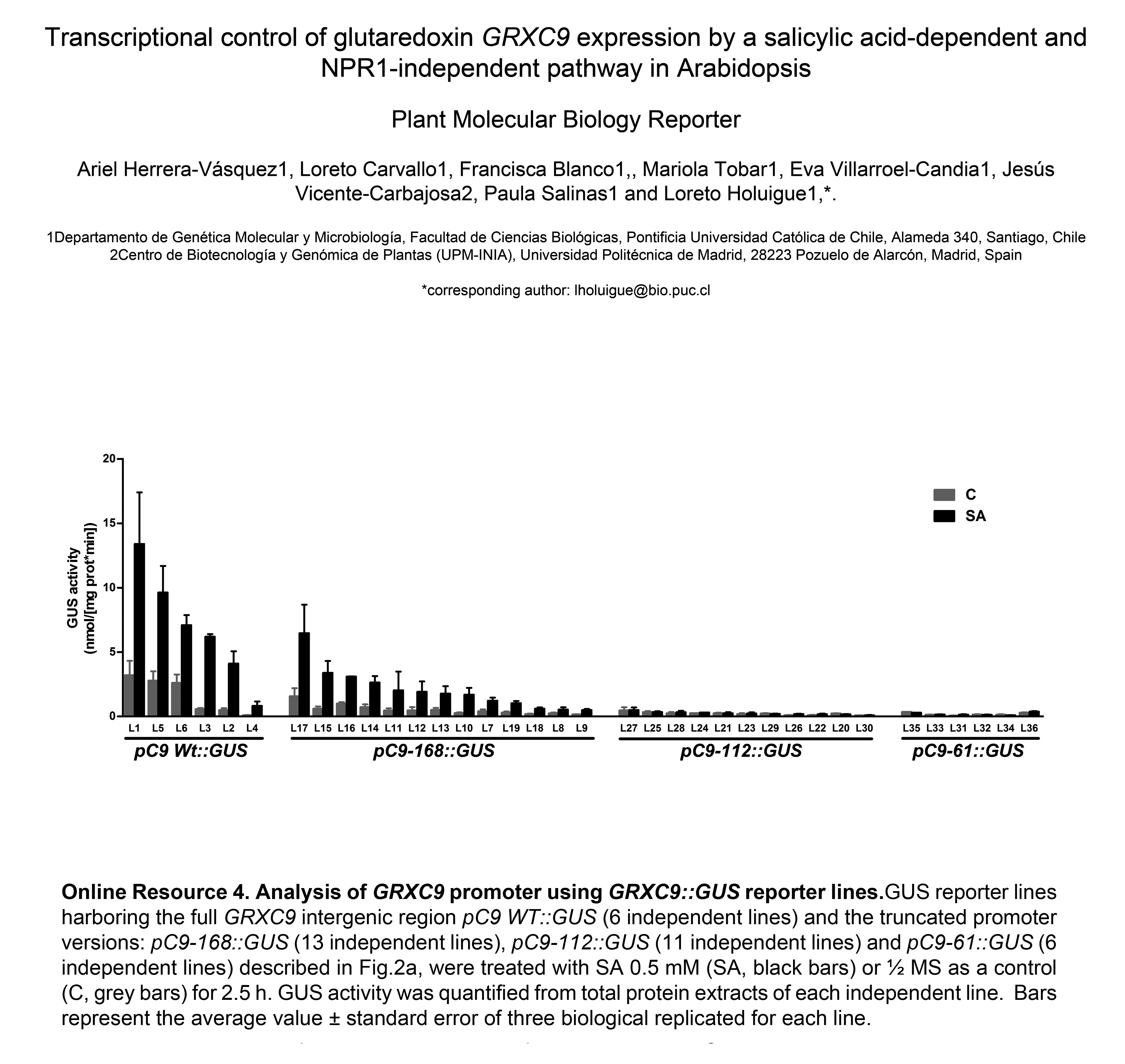

Supplement: Supplementary file 8 — High Resolution Image (TIFF 14914 kb) [file 11105_2014_782_MOESM4_ESM.tif]

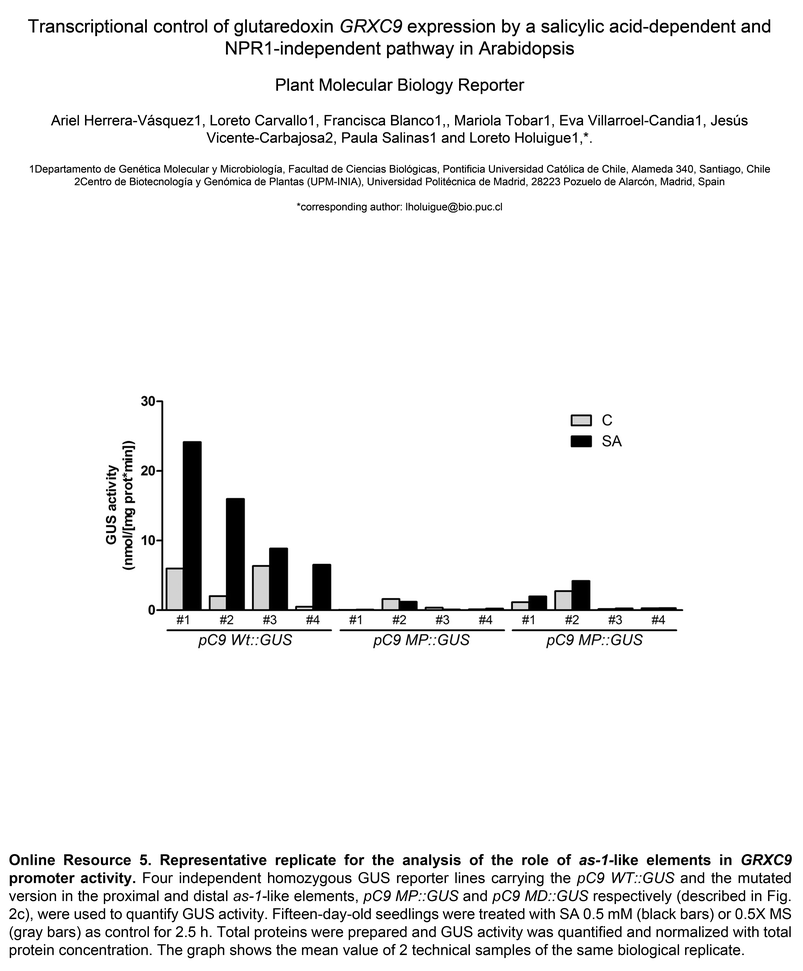

Supplement: Supplementary file 9 — (GIF 119 kb) [file 11105_2014_782_Fig12_ESM.gif]

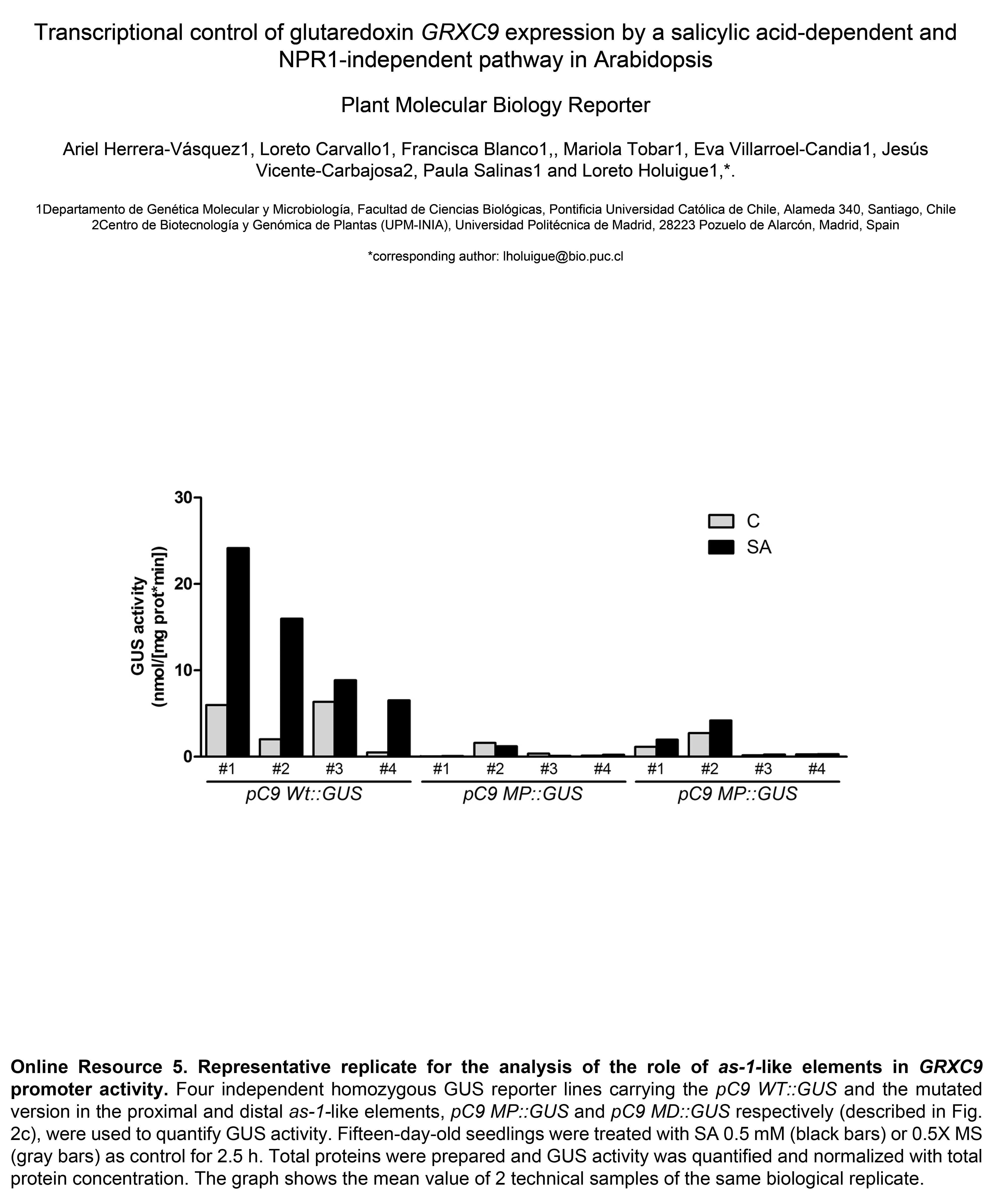

Supplement: Supplementary file 10 — High Resolution Image (TIFF 20470 kb) [file 11105_2014_782_MOESM5_ESM.tif]

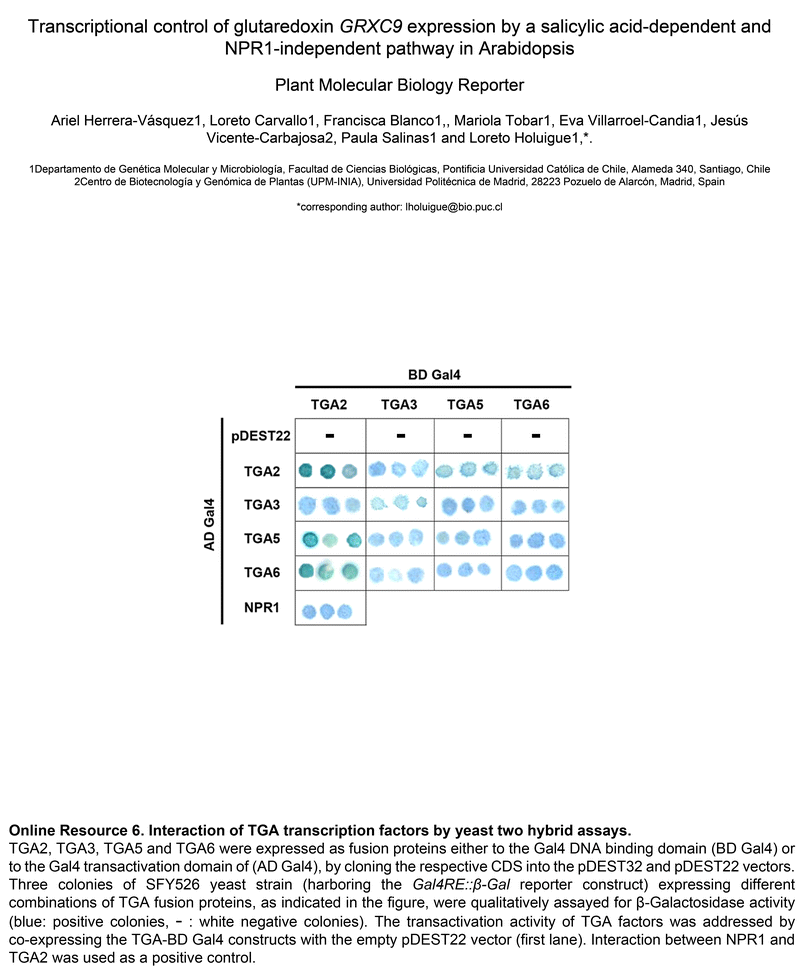

Supplement: Supplementary file 11 — (GIF 86 kb) [file 11105_2014_782_Fig13_ESM.gif]

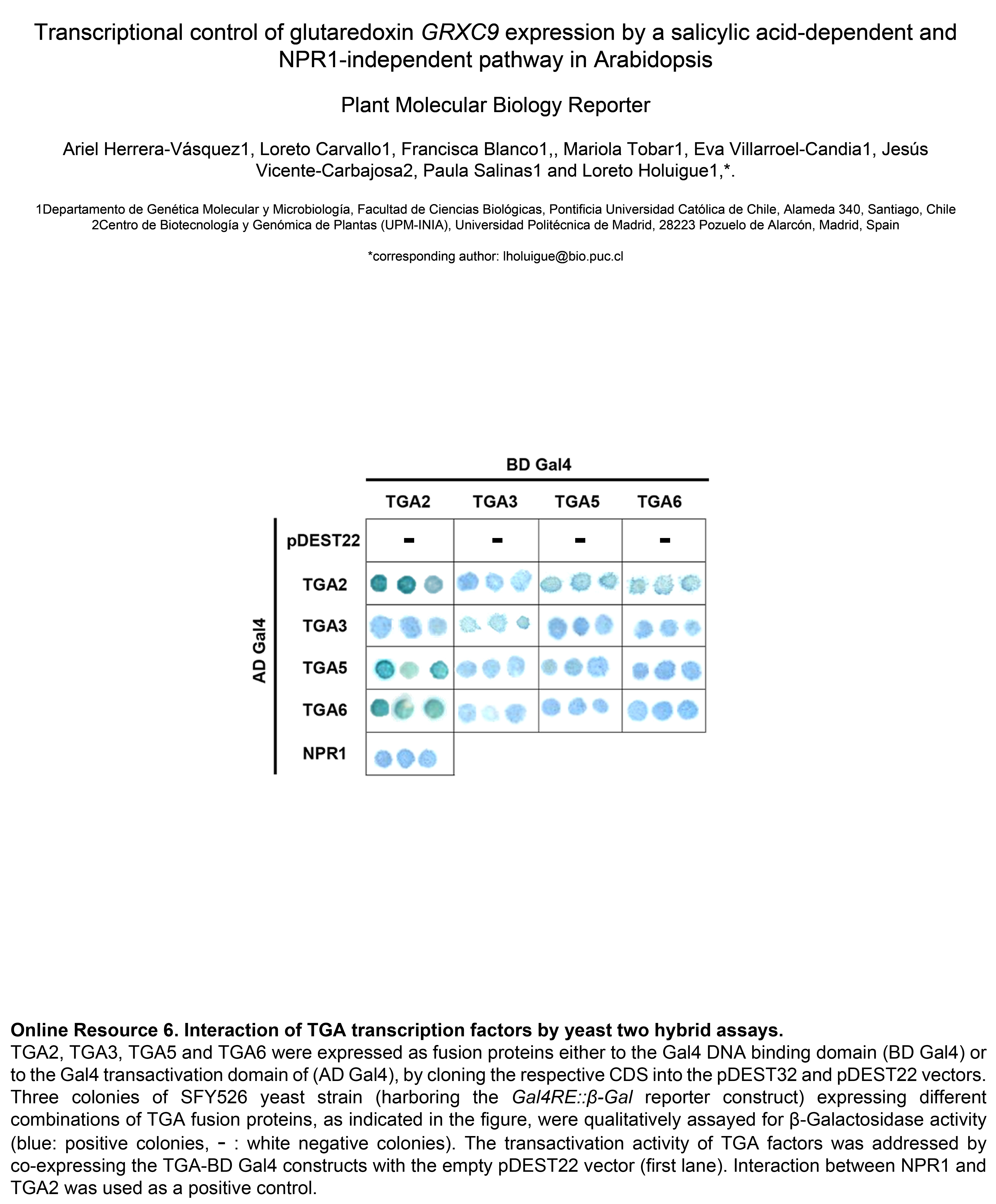

Supplement: Supplementary file 12 — High Resolution Image (TIFF 57136 kb) [file 11105_2014_782_MOESM6_ESM.tif]

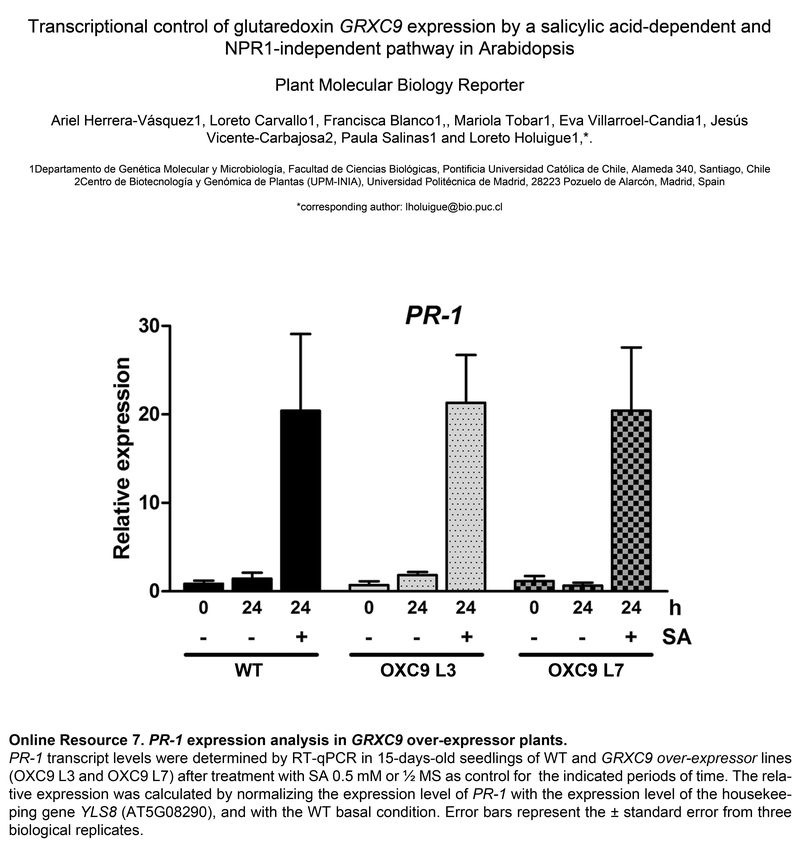

Supplement: Supplementary file 13 — (GIF 126 kb) [file 11105_2014_782_Fig14_ESM.gif]

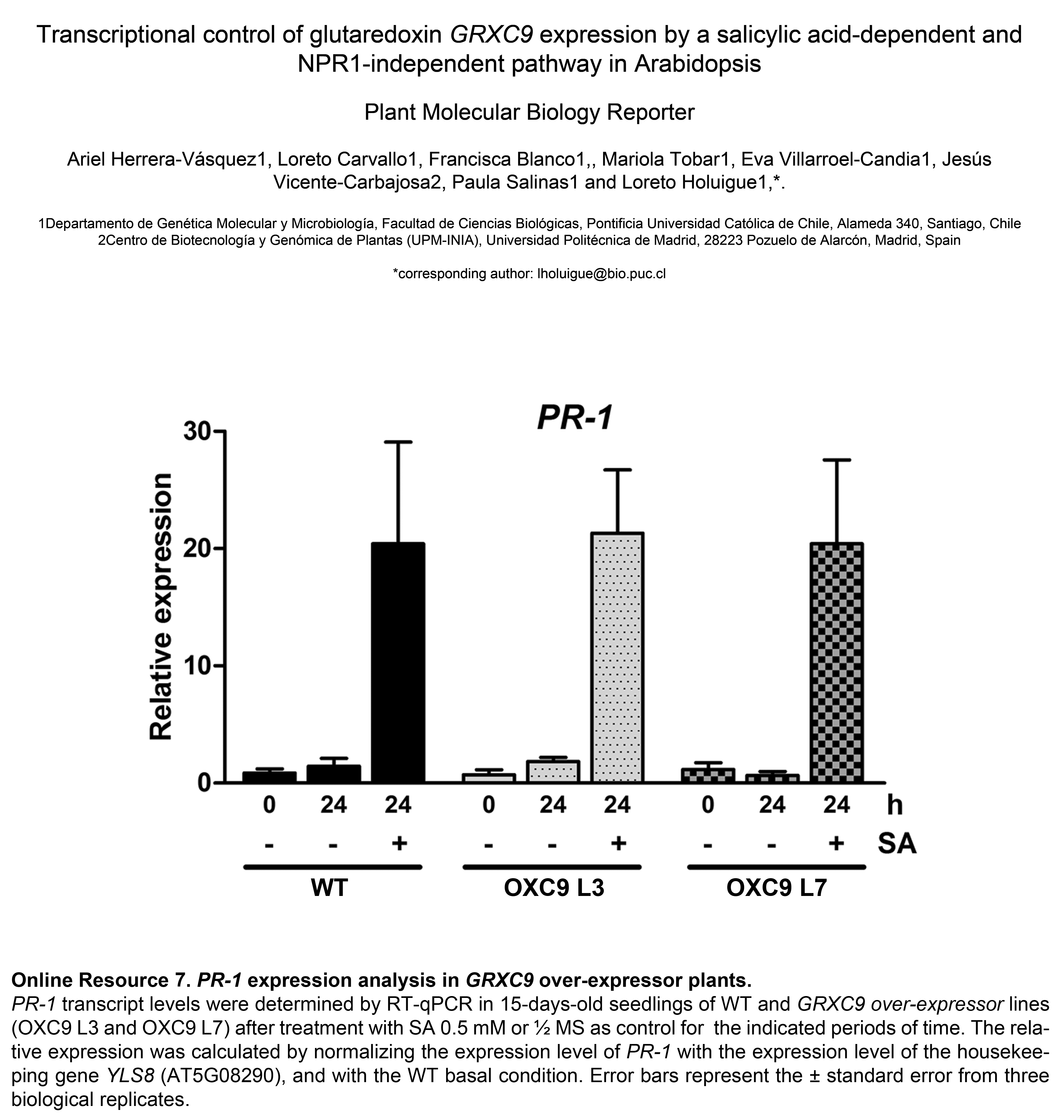

Supplement: Supplementary file 14 — High Resolution Image (TIFF 16613 kb) [file 11105_2014_782_MOESM7_ESM.tif]
