# Supplementary material for: Transcriptional Control of Glutaredoxin GRXC9 Expression by a Salicylic Acid-Dependent and NPR1-Independent Pathway in Arabidopsis
Source: Plant Mol Biol Report. 2014 Aug 14;33(3):624–37. doi: 10.1007/s11105-014-0782-5 (PMC4677692; doi:10.1007/s11105-014-0782-5)
Supplement: Supplementary file 15 — (DOCX 21 kb) [file 11105_2014_782_MOESM8_ESM.docx]

## Transcriptional control of glutaredoxin *GRXC9* expression by a salicylic acid-dependent and NPR1-independent pathway in *Arabidopsis*

**Plant Molecular Biology Reporter**

**Ariel Herrera-Vásquez^1,^ Loreto Carvallo^1^, Francisca Blanco^1,^**, **Mariola Tobar^1^**, **Eva Villarroel-Candia^1^, Jesús Vicente-Carbajosa^2^, Paula Salinas^1^ and Loreto Holuigue^1,*^.**

^1^Departamento de Genética Molecular y Microbiología, Facultad de Ciencias Biológicas, Pontificia Universidad Católica de Chile, Alameda 340, Santiago, Chile

^2^Centro de Biotecnología y Genómica de Plantas (UPM-INIA), Universidad Politécnica de Madrid, 28223 Pozuelo de Alarcón, Madrid, Spain

***Corresponding Author: lholuigue@bio.puc.cl**

**Online Resource 8.** Primers used for cloning genetic constructs, ChIP and RT-qPCR assays.

|  | | Target sequence | Forward Primer | | Reverse Primer | | |
| --- | --- | --- | --- | --- | --- | --- | --- |
| Cloning *GRXC9* promoter-GUS constructs | | | | | | | |
|  | | pC9 Wt | 5’ CACCAAAACGCATCACCTGC 3’ | | 5’ TTTCAAGTATGTTTTTAAAGATAG 3’ | | |
|  | | pC9 -168 | 5’ CACCGACACGGTCCTATG 3’ | | 5’ TTTCAAGTATGTTTTTAAAGATAG 3’ | | |
|  | | pC9 -112 | 5’ CACCCCATAGCTTCCTGTG 3’ | | 5’ TTTCAAGTATGTTTTTAAAGATAG 3’ | | |
|  | | pC9 -61 | 5’ CACCTTTCCTCTCTGATCTC 3’ | | 5’ TTTCAAGTATGTTTTTAAAGATAG 3’ | | |
| Cloning *GRXC9* CDS | | | | | | | |
|  | OX GRXC9 | | | 5’ CACCATGCCAAGGAACGATTTC 3’ | | 5’ CAACCACAGAGCCCCAACTTCCT 3’ |  |
| Site directed *CRXC9* promoter mutations | | | | | | | |
|  | | pC9 MD | 5’ TCCAATCCAGTTTTGTAAATAGC ACTATCACCCATAGCTTC 3’ | | 5’ GAAGCTATGGGTGATAGTGCTATTTAC AAAACTGGATTGGA 3’ | | |
|  | | pC9 MP | 5’ ATAGCTTCCTGTGTTTCACATCCTT ATTTAACCATCGTTGACG 3’ | | 5’ CGTCAACGATGGTTAAATAAGGATGTGA AACACAGGAAGCTAT 3’ | | |
| ChIP assays | | | | | | | |
|  | | GRXC9 proximal promoter -212 to +78 | 5´ GTGGGATCCAAAAAGTCAGC 3´ | | 5´ CGACGGTTGTCGTCATGTTA 3´ | | |
| RT-qPCR | | | | | | | |
|  | | *Total GRXC9*  (At1g28480) | 5’ CACTCCAAGTCCAAGAAGCAG 3’ | | 5’ AGAGAGTTCGGATGGTGGTG 3’ | | |
|  | | *Endogenous GRXC9*  (At1g28480) | 5´ TTAAGGAAGTTGGGGCTCTG 3´ | | 5’ CCGTAAACAACAATTACCAATCA 3´ | | |
|  | | *PR-1*  (At2g14610) | 5’ GTGGGTTAGCGAGAAGGCTA 3’ | | 5’ ACTTTGGCACATCCGAGTCT 3’ | | |
|  | | *CLATHRIN ADAPTOR COMPLEX* (At5g46630) | 5’ AATACGCGCTGAGTTCCCTT 3’ | | 5’ AGCACCGGGTTCTAACTCAA 3’ | | |
|  | | YLS8  (At5g08290) | 5´ TTACTGTTTCGGTTGTTCTCCATTT 3’ | | 5´ CACTGAATCATGTTCGAAGCAAGT 3’ | | |
